# Intracellular Protein Delivery: Approaches, Challenges, and Clinical Applications

**DOI:** 10.34133/bmef.0035

**Published:** 2024-01-25

**Authors:** Alexander Chan, Andrew Tsourkas

**Affiliations:** Department of Bioengineering, University of Pennsylvania, Philadelphia, PA, USA.

## Abstract

Protein biologics are powerful therapeutic agents with diverse inhibitory and enzymatic functions. However, their clinical use has been limited to extracellular applications due to their inability to cross plasma membranes. Overcoming this physiological barrier would unlock the potential of protein drugs for the treatment of many intractable diseases. In this review, we highlight progress made toward achieving cytosolic delivery of recombinant proteins. We start by first considering intracellular protein delivery as a drug modality compared to existing Food and Drug Administration-approved drug modalities. Then, we summarize strategies that have been reported to achieve protein internalization. These techniques can be broadly classified into 3 categories: physical methods, direct protein engineering, and nanocarrier-mediated delivery. Finally, we highlight existing challenges for cytosolic protein delivery and offer an outlook for future advances.

## Introduction

Over the past decade, protein biologics have consistently ranked among the top-selling drugs, accounting for nearly $300 billion in global sales annually [1]. Despite their popularity and immense therapeutic potential, proteins are generally not able to cross cell membranes. As a result, clinically approved protein therapeutics exclusively target surface-bound receptors or secreted antigens. Overcoming the membrane impermeability of proteins would dramatically expand the number of possible therapeutic targets and have direct benefits for human healthcare. Intracellularly targeted biologics would represent an exciting new class of drugs for many therapeutic areas including but not limited to oncology, infectious diseases, and genetic disorders.

## Comparison of Protein Delivery to Conventional Drug Modalities

Therapeutics sharing similar molecular features and mechanisms of action are often categorized into distinct modalities. Small molecules encompass the most common drug modality and are a cornerstone of modern medicine having several valuable properties including oral bioavailability, ease of manufacturing, and robust characterization methods. At less than 1 kDa in size, these chemicals can easily diffuse across cell membranes and produce rapid pharmacological effects. Protein biologics represent the next most-common drug modality. Comparatively, proteins are much larger than small-molecule drugs with molecular weights ranging from a few kDa to approximately 160 kDa in the case of antibodies. Recombinant proteins have soared in popularity owing to their structural complexity, allowing for highly specific receptor antagonism or agonism. Unlike small molecules, protein drugs exhibit poor oral bioavailability and are usually administered intravenously, intramuscularly, subcutaneously, or directly into target tissues. Despite this drawback, proteins are able to perform intricate biological functions inaccessible to small molecules, motivating their continued development in a wide breadth of therapeutic areas. Less commonly deployed biologics include nucleic acids such as DNA, messenger RNA (mRNA), and small interfering RNA (siRNA), viral vectors, and cell therapies. While relatively new, these therapeutic classes have shown promising outcomes in the clinic for the treatment of some previously intractable diseases, and their usage has expanded dramatically. For example, viral gene therapies have reversed the effects of inherited genetic disorders, and chimeric antigen receptor (CAR) T therapies have induced powerful regressions in aggressive and refractory blood cancers.

To date, many classes of drugs exist, and continued research efforts have accelerated the pace of therapeutics discovery. Still, each of these classes exhibit limitations stemming from their intrinsic chemical or pharmacokinetic/pharmacodynamic properties. Continued development of novel modalities is necessary to address the shortcomings of current drugs and address unmet medical needs. We compare cytosolically deliverable recombinant proteins to other drug classes and enumerate areas where this modality can tackle key issues facing modern pharmacology.

### Proteins versus small molecules: The undruggable proteome

Many proteins in nature possess relatively smooth surfaces and lack hydrophobic pockets making them difficult targets for traditional drug discovery, since small-molecule drugs require binding in functional or allosteric binding sites to exert their pharmacological effects. Thus, intrinsically disordered protein regions or targets lacking tractable active or allosteric binding sites remain refractory to traditional small-molecule drugs [2,3]. Pathogenic proteins not amenable to modulation by traditional small-molecule drugs due to localization and/or lack of binding sites are collectively known as “undruggable” or “under-drugged” targets. This so-called undruggable proteome is estimated to encompass 80% of known genes [4–7]. The proto-oncogene, KRAS (Kirsten rat sarcoma viral oncogene homolog), is one such example of an under-drugged target. Despite its established role as an oncogenic driver and 4 decades of concerted research efforts, only 2 small-molecule inhibitors have been approved for the treatment of RAS (rat sarcoma viral oncogene homolog)-mutant cancers [8]. Both of these inhibitors, sotorasib and adagrasib, target KRASG12C, which represents a minority of RAS mutations. The dearth of RAS inhibitors underscores an urgent need for new modalities able to modulate proteins eluding conventional pharmacological agents.

In stark contrast to small-molecule drugs, protein binders including antibodies [9,10], antibody fragments [11], and other single-domain scaffolds [12,13] can bind and inhibit virtually any target through protein–protein interactions (PPIs) spanning large interaction surfaces. Proteins can recognize targets in distinct conformational states or by site-specific post-translational modifications (PTMs). For example, many antibodies have been raised to discriminate proteins based on their phosphorylation [14], acetylation [15], and glycosylation [16] status. Importantly, proteins are also capable of binding intrinsically disordered regions [17], whereas small molecules require structured binding sites.

Owing to their exquisite specificity and high affinity against virtually any target, monoclonal antibodies (mAbs) have received considerable attention from the biopharmaceutical industry. Early proof-of-concept studies in the 1980s demonstrated that microinjection of RAS-inhibiting antibodies could block cell transformation, highlighting the enormous potential of protein biologics for inhibiting undruggable targets [18–20]. Currently, immunoglobulin G (IgG) drugs make up a large share of marketed therapeutics. This dominance is fueled by favorable biological properties and well-established discovery pipelines for new binders. Despite such benefits, current antibody therapeutics are restricted to surface-bound or circulating antigens, as their large size (>150 kDa) and hydrophilicity prevent entry into the cytoplasm. In the example of RAS-reactive antibodies, inhibition was only achieved by protein injection, an extremely low-throughput technique incompatible with clinical use.

Beyond full-length IgGs, other small protein scaffolds have emerged as antibody alternatives. One prominent example is the nanobody, a single-domain scaffold derived from the variable region of camelid antibody heavy chains. In a major step forward for non-mAb protein drugs, the Food and Drug Administration (FDA) approved the first nanobody, Caplacizumab, indicated for acquired thrombotic thrombocytopenic in 2019. Another promising small-protein scaffold is the Designed Ankyrin Repeat Protein (DARPin). Like nanobodies, DARPins are monomeric and amenable to high-yield, soluble expression using inexpensive *Escherichia coli* cultures. Many other small-protein scaffolds have also been developed including monobodies, affibodies, and affimers [12].

Currently, many therapeutic areas have unmet needs, as high-affinity drugs to modulate their pathogenic source do not exist. Even when small-molecule binders are discovered, their promiscuity can make them prone to off-target effects. Protein biologics address many of the pharmacological hurdles facing modern biopharmaceuticals but are generally restricted to extracellular applications. Gaining access to the cytosolic milieu would further expand the clinical utility of antibodies and other small binding proteins.

### Proteins versus viruses

As an alternative to the direct delivery of proteins into cells, viral vectors offer an opportunity to express the desired protein in infected cells. Viral vectors can produce long-lasting gene expression either by chromosomal integration or by episomal persistence of transgenes. Engineering of somatic cells may be particularly beneficial for the treatment of genetic disorders caused by an enzyme deficiency. This is bolstered by the numerous adeno-associated virus (AAV) therapies undergoing clinical trials for the treatment of monogenetic diseases [21]. However, many diseases do not require permanent modification, and transient delivery of therapeutics is often sufficient and even favorable, due to notable safety concerns with the use of viral vectors. In particular, viral vectors can trigger potent immune responses leading to severe adverse effects. For some biomedical applications, such as gene editing, sustained presence of gene editing enzymes could cause off-target editing and genotoxicity. As a result, viral vectors are only appropriate for a limited number of indications where a permanent genetic correction is needed. Otherwise, traditional drugs such as small molecules and proteins are more suitable and carry dramatically lower risks. For non-genetic diseases, protein delivery would be safer than viral vectors and allow titration of the therapeutic over the course of treatment.

### Proteins versus siRNA

Efforts to overcome undruggable targets have looked to protein knockdown rather than inhibition as a possible solution. The advent of RNA interference (RNAi) technology such as siRNA has enabled protein depletion via mRNA degradation. siRNAs are 21 to 25 nucleotide RNA molecules that hybridize with target mRNA and recruit RNA-induced silencing complexes to cleave complimentary transcripts [22,23]. Protein downregulation by siRNA has shown clinical success resulting in FDA approval of several breakthrough drugs [24–27]. Despite the promise of siRNA therapeutics, major limitations inherent to RNAi exist. Importantly, since siRNA knockdown is dependent on a protein’s intrinsic turnover rate, knockdown of stable protein targets can lag or outlast mRNA depletion [28–30]. This drawback is fundamental to the RNAi mechanism of action and can result in inefficient steady-state protein silencing. Additionally, off-target effects of siRNA are well-known and must be carefully considered for any siRNA drug [31–35].

By contrast, proteins can be selected for their high affinity and selectivity, decreasing the risk for off-target effects. Moreover, protein-based inhibitors and degraders have more predictable kinetics compared to siRNA, as they directly modulate the target protein. Unlike siRNA, protein-based drugs can also discriminate between distinct PTM subpopulations. Finally, protein inhibitors can block specific interfaces on target proteins, while avoiding others, which could allow for more precise control over the cellular response.

### Proteins versus mRNA and DNA

Therapeutic proteins may be produced in target cells themselves from transfected DNA or RNA vectors encoding the proteins of interest. Plasmid DNA is commonly used for high-level exogenous expression of proteins in cell culture. Following DNA transfection, plasmids first translocate to the nucleus where mRNA is transcribed. The nascent mRNA is exported to the cytosol and translated into protein. Alternatively, mRNA can be directly transfected into cells to obviate transcription, allowing for immediate protein translation in the cytosol. Direct comparisons between DNA and RNA protein expression kinetics reveal that mRNA-mediated protein expression is rapid and homogeneous, while protein expression from transfected DNA is both slower and more heterogeneous [36]. Another functional distinction between these 2 nucleic acids methods is that cytosolically delivered mRNA has no risk of genomic integration, whereas DNA poses a small chance of random insertion. Owing to fast kinetics and transience, mRNA is regarded as the more viable therapeutic modality of the two. The advantages of mRNA were demonstrated with the deployment of both the Moderna and Pfizer/BioNTech COVID-19 vaccines [37,38] comprising SARS-CoV-2 spike protein encoding mRNA encapsulated within ionizable lipid nanoparticles (LNPs). Despite these benefits, mRNA drugs suffer from poor enzymatic stability and inherent immunogenicity. To prevent mRNA degradation, care must be taken to avoid nucleases at all stages of production and storage. The innate immunogenicity of mRNA must be tempered by substitution of uridine with non-immunogenic isomers [39,40].

Compared to nucleic acids, direct cytosolic protein delivery offers an even more rapid therapeutic modality than both DNA and mRNA. Cytosolically delivered proteins would be immediately functional, bypassing nuclear translocation, transcription, translation, and folding steps required with nucleic acid methods. Similar to mRNA, intracellularly delivered proteins would be transient in nature, degrading via endogenous lysosomal and proteasomal pathways. While protein stability varies, proteins are generally more stable than mRNA, both in storage and in biological media [41]. Finally, it has been reported that some therapeutically interesting proteins are poorly expressed as transgenes despite facile purification from *E. coli* cultures [42]. Thus, for some applications, nucleic acids may be precluded entirely as a treatment modality, and direct protein delivery could be the only method for introducing potent therapeutics into the cytoplasm.

## Therapeutic Applications of Intracellular Protein Delivery

Intracellular delivery of proteins can fulfill nearly all the biomedical functions currently afforded by current modalities including small molecules, nucleic acids, and viral vectors. One of the most common ways drugs modulate biological responses is by antagonizing effectors and blocking their biochemical functions. In these applications, proteins can function as potent stoichiometric inhibitors, providing greater specificity than most small-molecule drugs. One area where this high-affinity binding can be leveraged is in cancer therapy where many promising targets have been identified for precision therapy but lack efficacious inhibitors. For event-driven pharmacology, catalytic activity can be achieved with direct delivery of metabolic enzymes or proteases. In the treatment of genetic disorders, programmable nucleases can be delivered for long-term genetic correction. Other therapeutic applications pursued for protein drugs include vaccines, where antigen delivery can invoke long-term immunity, and targeted protein degradation, by exploiting the strong binding affinity of proteins against undruggable targets. Finally, proteins may be particularly advantageous compared to other modalities in the treatment of diseases requiring immediate intervention, such as sepsis and septic shock, where patient health can quickly deteriorate, and survivability is highly dependent on rapid medical action.

### Cancer

The advent of small-molecule chemotherapeutics ushered in a new era for cancer treatment by eliminating rapidly dividing tumor cells. Similarly, proteins inhibiting essential cellular functions offer a direct method to kill cancer cells and suppress tumor growth. Proteases [43,44], bacterial toxins [45], and apoptosis inducers [46] are promising candidates for cancer therapeutics. Saporin is a highly active inhibitor of protein synthesis and of high interest for potent suppression of tumor cells [47]. However, saporin must be inside of target cells to inhibit ribosomes. The membrane impermeability of cytotoxic proteins has limited their conversion into successful cancer therapies. Just as small-molecule chemotherapeutics enabled generalized tumor inhibition, successful intracellular protein delivery would potentiate new therapeutic modalities for tumor inhibition. To this end, drug delivery researchers have worked to deliver saporin (and other cytotoxic proteins) into tumor cells in the hopes of producing potent next-generation cancer therapeutics.

Precision therapy is another method to inhibit tumor growth by targeting specific pathway effectors known to drive proliferation. The ability to inhibit intracellular targets with high specificity is especially important in oncology, as intricate signaling pathways are known to orchestrate cancer initiation, maintenance, and metastasis. Some of the most well-known proto-oncogenes are p53, RAS, and MYC. Mutations in RAS-family proteins activate both the canonical RAS/RAF/MAPK and PI3K/AKT/mTOR pathways, inducing cell proliferation and survival in transformed cells. Likewise, overexpression of MYC causes transcriptional dysregulation and cancer pathogenesis. Mutant p53 leads to tumorigenesis via inactivation of the protein’s endogenous tumor-suppression functions. Collectively, dysregulation of RAS, p53, and MYC genes are frequently found in many types of cancers. It is estimated that RAS and p53 mutations are present in approximately 30% and 50% of cancers, respectively, while the MYC amplification rate in cancers is estimated to be 21% [48]. Targeting the highly prevalent aberrant forms of these genes is highly attractive for cancer therapy, as they play essential roles in cancer pathogenesis.

Despite their established role in cancers, p53, RAS, and MYC are widely regarded as undruggable due to a lack of hydrophobic binding pockets. In recent years, progress has been made toward drugging KRASG12C mutants with FDA-approved covalent inhibitors, but pan-KRAS inhibitors remain elusive [8]. Likewise, several MYC and P53 modulators are under investigation but have not been approved by the FDA [48,49]. Unlike small molecules, proteins have no difficulty binding these same targets. For example, extensive screening and selection of small-protein scaffold libraries have yielded low nanomolar to picomolar binders including several potent inhibitors of high-priority oncogenic targets [50–53]. Notably, DARPinK27 [54] and DARPinK19 [55] are potent pan-RAS and pan-KRAS inhibitors, respectively. In addition, OmoMYC is a miniprotein inhibitor of MYC [56]. Finally, antibodies with the potential to inhibit undruggable targets have been identified [57,58]. With an effective cytosolic delivery method, these proteins can be converted into potent precision therapeutics to overcome undruggable oncogenes.

### Gene editing

The discovery and refinement of CRISPR/Cas gene editing systems has revolutionized the life sciences and catalyzed breakthroughs in cell and gene therapies [59–62]. The CRISPR/Cas9 system is capable of producing double-stranded breaks at defined genomic locations for gene knockout or knock-in (with the addition of homologous template DNA). It is a highly valued genetic engineering tool due to its precision, programmability, and ease of implementation. Most recently, base editors and prime editors have been developed from catalytically inactivated Cas for precise single-nucleotide edits, insertions, and deletions without the need for double-stranded breaks [63,64]. For all Cas-based molecular tools, successful delivery and nuclear transport of functional enzymes are prerequisites for genome engineering. For ex vivo gene-editing applications including engineering cells for adoptive cell transfer, electroporation of Cas ribonucleic proteins (RNPs) typically suffices. However, electroporation causes cytotoxicity and yield loss [65]. Viral vectors are also commonly employed to express Cas9 gene editing components in target cells. However, immunogenicity, handling risks, and lack of control over gene expression with viral vectors remain major concerns [66,67]. Critically, persistent expression of Cas9 by viral vectors is associated with increased off-target editing, highlighting the importance of brief Cas9 activity [68]. Nucleic acid transfection including plasmid DNA and mRNA has been pursued as alternatives to viruses and electroporation, but they suffer from additional challenges discussed above. Due to the pitfalls of both viral and nucleic acid methods alike, cytosolic delivery of whole Cas proteins is an attractive alternative, offering safe, rapid, and transient gene editing.

### Targeted protein degradation

Proteolysis-targeting chimeric molecules (PROTACs) have recently emerged as a promising method for post-translational degradation of intracellular targets. The classical PROTAC is a heterobifunctional molecule comprising a protein-binding “warhead” and an E3-recruiting ligand joined by a chemical linker. PROTACs simultaneously bind a protein of interest (POI) and an E3 ligase. Through a multi-step enzymatic process, ubiquitin proteins are attached to the POI by virtue of proximity to the E3 ligase. Ubiquitinated POIs are marked for destruction by cells’ endogenous ubiquitin-proteasome system (UPS), bypassing the need for functional inhibition. Since their inception in 2001 [69], PROTACs have gained tremendous traction as a viable therapeutic class.

Despite the advantages of protein degradation over inhibition, PROTAC discovery is limited by the availability of target-binding molecules. Even without the requirement of binding an active/allosteric site, binders for undruggable targets are lacking [70]. When high-affinity binders do exist, linker optimization remains a major bottleneck to PROTAC development [71]. The small length scale of traditional PROTACs introduces many new interactions between the POI and E3 ligase residues at the POI-PROTAC-E3 interface. These neo-PPIs can destabilize the POI-PROTAC-E3 ternary complex and impede ubiquitination. Often, empirical linker engineering is the only way to overcome this issue.

As an alternative to small-molecule PROTACs, bioPROTACs are analogous protein-based bi-functional degraders and offer an attractive method for targeted protein degradation while requiring little optimization [72]. By mimicking natural E3 ligases, bioPROTACs obviate the need for linker screening, since the POI and E3 domains are sufficiently separated to avoid steric clashes. In a recent example demonstrating the potential of biologics-based degraders, cytosolic delivery of target-specific IgGs in Tripartite motif containing-21 (TRIM21)-expressing cells was shown to induce degradation of diverse protein substrates [73]. This technique, called TRIM-Away, enabled near-complete elimination of various targets including the kinases extracellular signal-regulated kinase (ERK) and inhibitor of κB (IκB) as well as stable proteins such as green fluorescent protein (GFP) and Rec8. TRIM-Away displayed rapid degradation kinetics with target half-lives as fast as 10 min and was achieved using readily available antibodies. Currently, most bioPROTAC systems, including TRIM-Away, are either expressed in cells as genetic constructs or delivered by microinjection or electroporation, severely limiting their therapeutic utility. To fully realize the potential of bioPROTACs, efficient methods for protein delivery must be developed. Efficient cytosolic delivery of recombinant, exogenous bioPROTACs would greatly expand the arsenal of targeted protein degraders and address the shortcomings inherent to traditional small-molecule PROTACs.

### Enzyme replacement therapy

Protein delivery would be highly applicable in the treatment of diseases caused by an enzyme deficiency, as direct intracellular delivery of enzymes would immediately restore cellular homeostasis and alleviate symptoms. One therapeutic area where enzyme replacement therapy can address unmet medical needs is in the treatment of urea cycle disorders. In urea cycle disorders, a deficiency in 1 of 6 urea cycle enzymes blocks the conversion of ammonia into urea, a benign metabolite filtered by the renal system for urine excretion. Dysfunction of the urea cycle causes a buildup of toxic ammonia in the bloodstream. Hyperammonemia causes a host of symptoms including lethargy and neurological impairment [74]. Currently, treatment options for urea cycle disorders are limited, and chronic manifestations of the disease are typically managed by ammonia scavengers, dialysis, and reducing protein intake. However, none of these options address the root cause of hyperammonemia. Delivery of functional enzymes may be one avenue for treating urea cycle disorders at the source by restoring liver cells’ ability to break down ammonia.

### Vaccines

In response to an infection, the body’s antigen-presenting cells (APCs) engulf and digest pathogens, displaying its components on the cell surface. This activates the adaptive immune response, enabling the body to fight future infections via both T-cell and antibody-mediated responses. On their own, purified antigens are poorly immunogenic, and nanoparticle delivery systems have been explored to promote a stronger immune response. This is accomplished by improving both antigen uptake and the nanomaterials themselves acting as immunostimulatory adjuvants [75]. Naturally, such delivery systems can be developed for any diseases currently treated with standard vaccines, including viral and bacterial infections. In recent years, anti-cancer vaccines have also been proposed by delivering tumor-specific neo-antigens to dendritic cells [76]. In this immunotherapy approach, cancer-specific proteins and peptides are used to train immune cells to kill tumor cells. However, tumor-specific antigens are less defined and more weakly immunogenic compared to bacterial and viral antigens, and the uptake mechanisms utilized by APCs, namely, receptor-mediated endocytosis and pinocytosis, result in poor tumor antigen processing and insufficient immunity [77]. Methods to enhance cytosolic protein delivery into APCs is one approach to enhance tumor antigen presentation and potentiate strong anti-tumor immune responses.

### Sepsis

Sepsis is a potentially fatal syndrome caused by a dysregulated inflammatory reaction to microbial infection. During sepsis, both pro-inflammatory and anti-inflammatory responses are activated leading to fever, tachycardia, endothelial damage, and improper clotting [78]. In some cases, sepsis can progress into septic shock, a severe condition characterized by a dangerous drop in blood pressure and multiple organ failure. Septic shock is a life-threatening condition with mortality rates approaching 50% to 60% [78,79]. During the acute phase, sepsis symptoms are initiated by the secretion of pro-inflammatory cytokines mere minutes after immune recognition of pathogen-associated molecular patterns and damage-associated molecular patterns. To combat sepsis, an equally prompt treatment response is necessary to improve chances of survival. Typically, patients are administered broad-spectrum antibiotics and intravenous fluids at the first signs of disease. Treatment delay of just a few hours can dramatically worsen prognosis for patients experiencing septic shock [80].

Central to the rapid onset of sepsis symptoms is the upregulation of “early activation genes” interleukin (IL)-1, IL-12, IL-18, tumor necrosis factor (TNF)-α, and interferon (IFN)-γ. Many of these inflammatory signals are mediated by the activation, nuclear translocation, and increased transcriptional activity of nuclear factor-κB (NF-κB) [81]. Blockade of NF-κB pathway signaling is a promising option to effectively stem patient deterioration. For the treatment of sepsis and septic shock, protein delivery is especially well-suited for 2 reasons. Firstly, NF-κB remains undruggable with conventional modalities [82]. Small-molecule inhibitors have been developed for upstream pathway effectors, but these agents are toxic and have not progressed beyond phase 2 clinical trials [83]. Secondly, protein delivery is the fastest way to introduce biologics intracellularly for target modulation. In sepsis, where survival depends on timely intervention on the order of hours, methods to quickly block NF-κB signaling are crucial to ameliorate symptoms. Importantly, while systemic inhibition of NF-κB is associated with on-target toxicities owing to the transcription factor’s ubiquitous expression [82], the short treatment window needed for septic shock management would limit such adverse effects and poses an important use-case for NF-κB inhibition.

## Methods for Intracellular Protein Delivery

**Fig. 1.**
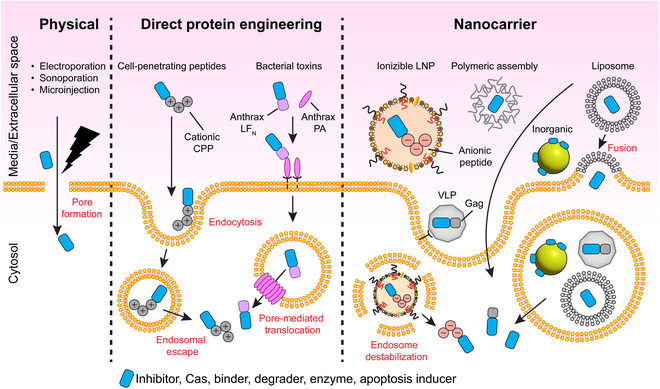
Overview of common intracellular protein delivery approaches. Physical approaches involve mechanical disruption of the cell membrane and includes techniques such as electroporation, sonoporation, and microinjection. Direct protein engineering approaches rely on fusion/bioconjugation to transduction domains originally derived from viral and bacterial proteins. Nanocarrier systems have been developed for protein delivery using diverse materials from synthetic lipid nanoparticles and polymeric supramolecular assemblies to bioinspired VLPs*.*

Most reported techniques for intracellular protein delivery can be broadly classified into 3 categories (Fig. 1). Physical methods are the most direct way to deliver native recombinant proteins without any chemical or genetic modifications. These methods involve physically perturbing target tissues/cells to take up proteins in solution. Proteins can also be directly engineered with targeting and/or transduction moieties for enhanced delivery. Lastly, proteins may be shuttled across cell membranes using synthetic or biological nanocarriers. Nanocarrier methods can be used to deliver either native proteins or modified proteins.

Many of the technologies described below were originally developed for the delivery of small-molecule drugs or nucleic acids. We focus on how these intracellular delivery systems were adapted for proteins through modifications to the delivery system and/or the cargo itself. One benefit of using protein cargo is the ability to modify their behavior either by bioconjugation, by changing their amino acid sequence, or by fusing functional domains to generate chimeras. For example, recombinant proteins can be directly fused to delivery motifs via flexible, glycine- and serine-rich linkers. In addition, proteins can be functionalized with additional cargo (e.g., small-molecule drugs or imaging agents) using one of a myriad of existing chemistries exploiting reactive amino acid side chains. Typically, chemical bioconjugation occurs at cysteine residues’ sulfhydryl groups or at lysine residues via its primary amine. Enzymatic methods for post-translational protein conjugation utilizing sortase or tyrosinase are also available [84].

### Physical methods for cytosolic protein delivery

Physical disruption of the cellular membrane is the most direct method to introduce exogenous proteins into cells. Temporary pores can be created in cell membranes by an electrical current [85], focused ultrasound [86], or squeezing through microfluidic channels [87], permitting proteins to freely cross into the cytosol. Proteins can also be directly introduced into the cytosol by microinjection [88,89]. Physical delivery methods are best suited for ex vivo applications such as adoptive cell transfer where one-time delivery of genome editing enzymes produces permanent changes.

Electroporation is a common strategy to introduce Cas9 RNPs, base editors, and prime editors into patient-derived T cells to enhance CAR T cell function and improve cancer immunotherapy. Similarly, membrane deformation using microfluidic devices has been reported for intracellular delivery of RNPs [90], although this approach is less common. Physical protein delivery methods can also serve to accelerate proof-of-concept studies for promising new therapeutic modalities. Recently, the TRIM-Away method for targeted protein degradation was introduced, requiring cytosolic delivery of a target-specific IgGs and TRIM21, an antibody-directed E3 ligase. To dissect the biological mechanisms behind TRIM-Away for targeted protein degradation, Clift et al. [73] used microinjection and electroporation to guarantee rapid delivery of proteins into the cytosol. In another study, Ibrahim et al. [91] showed that a recombinant nanobody-based bioPROTACs could be delivered by electroporation for degradation of fluorescent fusion proteins.

### Direct engineering of proteins for cytosolic delivery

While most proteins cannot cross the cell membrane, some peptides and proteins do have an intrinsic ability to enter cells by direct translocation, endocytosis, or micropinocytosis. Most of these peptides are derived from viral and bacterial virulence factors having evolved over time to invade and infect mammalian host cells. Fusion of cargo proteins with these cell-permeant peptides and proteins constitute some of the most-studied methods for cytosolic protein delivery.

#### Cationic protein transduction domains

In the late 1980s, viral proteins were found to possess an intrinsic ability to cross cell membranes [92], sparking interest in a potential drug delivery modality. Intracellular protein therapeutics could be made possible via these “cell-penetrating peptides” or CPPs. Fueled by promising proof-of-concept results, CPP sequences have been extensively explored for cytosolic delivery of therapeutic biomolecules including small molecules and nucleic acids as well as larger cargo such as nanoparticles. The first CPPs discovered were linear peptides derived from viruses [93]. Notably, the Trans-Activator of Transcription (TAT) protein is an 81-amino-acid protein critical to the lifecycle of Human Immunodeficiency Virus (HIV). The polybasic region of HIV TAT (amino acids 37 to 72) assists uptake of viral machinery into human leukocytes. Early work demonstrated that TAT could be conjugated to membrane-impermeable proteins to induce cellular uptake of cargo including β-galactosidase, horseradish peroxidase, and Fab domains [94,95]. Internalization by TAT fusion is mediated by polycationic charges imbued by lysine (K) and arginine (R) residues in the CPP [96]. Other popular cationic CPPs include penetratin [97] and polyarginine [98,99].

Originally, CPPs were directly conjugated to proteins for delivery. However, in 2001, Morris et al. [100] demonstrated that CPP-mediated intracellular protein delivery was possible without the need for covalent coupling using a novel CPP dubbed Pep-1. This designed CPP contained 3 domains: a hydrophobic tryptophan-rich domain for protein–CPP interaction and complexation, a hydrophilic SV-40-derived lysine-rich domain for electrostatic interaction with cell membranes, and a flexible spacer between the 2 hydrophobic and hydrophilic sequences. The amphipathic Pep-1 peptide could be complexed with cargo proteins via simple mixing of the 2 components. By eschewing genetic fusion or bioconjugation, Pep-1-mediated delivery could be easily optimized by tuning the molar ratio between CPP and payload protein. In addition, delivery was efficient for model fluorescent proteins. GFP was detectable in >60% of cells following incubation with just 50 nM GFP mixed with 500 nM Pep-1.

Non-traditional cationic transduction domains have also been reported for improved protein transfection efficiency compared to earlier short, linear CPPs. Cronican et al. [101] fused red-fluorescent mCherry to supercharged +36GFP and demonstrated a 20- to 100-fold increase in protein internalization compared to TAT, R10, and penetratin. Yin et al. [102] generated well-expressed, highly charged transduction sequences by screening long, lysine-containing polypeptides for soluble expression in bacterial cultures. Compared to commonly used CPPs typically having less than 20 amino acids, these transduction domains were as large as 360 amino acids and had theoretical charges as high as +60. The optimal polypeptide identified, K4, had a +40 theoretical charge, contained 120 amino acids, and demonstrated superiority compared to both TAT and R10. Genetic fusion with K4 promoted efficient delivery of proteins spanning isoelectric points ranging from 3.7 to 10.6 as well as molecular weights ranging from ~10 to 160 kDa. Invariably, K4 redirected proteins to the nucleus when fused to cargo proteins and demonstrated utility as a Cas9 delivery tool. In a later study, Wang et al. used a similar methodology to identify a 40-amino-acid CPP with +20 charge for cytosolic protein delivery [103]. The lead peptide, K20, could be used either as a genetic fusion or as a protein transfection reagent for native protein delivery when a phenyl boronic acid residue was incorporated into the CPP.

#### Cyclic CPPs

Cyclic CPPs were born from advancements in cyclic peptide research. They are more resistant to proteolytic cleavage and can improve cell membrane binding [104]. Qian et al. [105] developed a library of cyclic CPPs containing a combination of both hydrophobic and cationic residues. From this library, a lead cyclic CPP, cFΦR4 (Φ = L-2-naphthylalanine) was identified, displaying more efficient cellular association and uptake compared with the linear version, FΦR4. The amphipathic cFΦR4 associated with cells 13 times more efficiently than the linear R9 cationic CPP, and intracellular uptake of cFΦR4 was 200% more than R9 [105]. Later analogues cFΦR4 showed even greater cell penetration with analogues displaying 600% improvement over the original cyclic CPP [106]. Other groups have demonstrated similar advantages of cyclic CPPs as well. Nischan et al. [107] showed that cyclized TAT conjugated to GFP by copper-catalyzed azide-alkyne click chemistry improved cellular uptake of cargo protein by 2 orders of magnitude relative to linear TAT. Schneider et al. [108] delivered mCherry protein with cyclic R10 and further showed that a cleavable disulfide bond between cargo and the CPP could tune intracellular localization of the mCherry molecule. Cyclic R10 was also harnessed for delivery of both nanobodies as well as Mecp2, a protein deficient in patients with Rett syndrome [109].

#### Multimeric CPPs

One approach to further improve CPP-mediated delivery is by designing multimeric peptides that increase the number of transduction moieties per cargo molecule. Multiple groups have reported improved protein transfection with multimeric versions of both linear and cyclic TAT compared to their monomeric counterparts [110,111]. Structure-defined multimeric CPPs have also been explored. In one example, Oh et al. designed leucine (L) and lysine (K) amphipathic CPPs (LK peptides), which form defined helix-loop-helix (HLH) motifs analogous to natural HLH structures found in endogenous proteins [112]. Starting from the unit peptide, LK-1 (LKKLLKLLKKLLKLAG), flexible diglycine linkers were added between tandem repeats of the sequence generating multimeric CPPs comprising antiparallel helices. Increased delivery was observed as a function of LK repeat number up to LK-4. Importantly, dose-dependence uptake studies performed on LK-4 fused to GFP demonstrated fluorescence in 80% of cells at just 200 nM protein. Thus, multimeric amphipathic CPPs hold promise for protein cargo delivery at low concentrations. In another example, Iwata et al. [113] conjugated 3 monomers of an attenuated cationic lytic peptide, L17E, to an Fc-binding peptide to produce trimeric FcB (L17E)_3_. Conceived to specifically enhance IgG delivery, FcB (L17E)_3_ was found to also enable the transfection of other anionic proteins including -30GFP, -30GFP-conjugated anti-mCherry-nanobody, and AlexaFluor488-conjugated IgGs. The multimeric FcB (L17E)_3_ peptide displayed significantly higher cargo internalization at lower CPP amounts compared to the L17E monomer. Moreover, FcB (L17E)_3_ exhibited a distinct mode of action in which the CPP formed liquid droplets with protein cargo. When these liquid droplets came in contact with cell membranes, protein cargo rapidly diffused across the cell within seconds. Similar to FcB (L17E)_3_, histidine-rich beak peptides (HB*pep*) can also undergo liquid–liquid phase separation to form peptide coacervates. Sun et al. generated disulfide-modified HB*pep* variants that spontaneously phase separate at neutral pH, capturing protein cargo into liquid droplets [114]. Critically, these droplets can directly cross the cell membrane via a non-endocytic mechanism. Upon cell entry, the peptides are reduced by cytosolic glutathione and droplets disassemble, rapidly releasing protein cargo into the cell. At low micromolar concentrations, the proteins EGFP, R-phycoerythrin, lysozyme, BSA, saporin, and β-galactosidase were successfully transfected into cells with delivery efficiencies approaching 100%.

#### CPPs for antibody delivery

Several cationic and amphipathic CPPs can be utilized for antibody delivery by simple complexation of IgG payload with CPPs [100,103,115]. However, IgG delivery via non-covalent interactions with these peptides raises some concerns. Firstly, while some amphipathic CPPs are seemingly agnostic to protein payload size [116], others have reported decreased cellular uptake for large protein cargo such as IgGs (~160 kDa) [117]. In addition, amphipathic CPPs may bind randomly to the IgG surface during complexation and mask the Fab region, decreasing target affinity. Due to the limitations of classical CPPs for antibody delivery, more sophisticated and IgG-tailored CPP technologies have been developed.

In one example, Mie et al. [118] engineered a TAT-B2C fusion protein where B2C contains the IgG-binding B domain derived from *Staphylococcus aureus* protein A. Full-length IgG was mixed with purified TAT-B2C and successfully delivered into 3T3 cells. The TAT-B2C protein showed saturated delivery at 30 μM. In another study, Gaston et al. [119] fused 6 different CPPs: TAT, Pep-1, PEPth, aurein, MTS, and GFWFG sequences to 3 different regions of an anti-CEACAM5 antibody to investigate optimal designs for antibody–CPP fusions. Placement of CPPs at either the light-chain N-terminus or at the heavy-chain N-terminus resulted in low expression yields, and delivery was found to be favored when CPPs were fused at the hinge region. Of the 6 tested CPPs, both Pep-1 and PEPth showed appreciable cytosolic delivery following treatment with CPP-fused antibody at 2 μM for 24 h, albeit at low efficiencies (<5%). While promising as a proof-of-concept study, the modest intracellular delivery exhibited in this study by genetic fusion could severely limit this method for cytosolic antibody therapy. Furthermore, uptake efficiency was dependent on the CEACAM5 expression level, suggesting that antibody–antigen recognition is a prerequisite for some CPP-based antibody delivery platforms. In another study, Sauter et al. [111] synthesized tetrameric CPPs (tCPP) with α- and ε-Fmoc-protected lysine building blocks as branching points and a cysteine focal point serving as the antibody crosslinking moiety via sulfo-SMCC chemistry. Eight different CPPs were synthesized as tCPPs and conjugated to the humanized anti-EGFR antibody, Matuzumab. Cationic tCPPs including tetrameric-TAT and tetrameric-R9 demonstrated the highest delivery rates ranging from 37% to 46%, whereas tetramer versions of amphipathic CPPs were internalized less efficiently at less than 32% delivery efficiency. When compared head-to-head against monomeric TAT, tetrameric TAT displayed a modest increase in antibody translocation. Encouragingly, it should be noted that tCPPs exhibit cellular uptake of antibodies at nanomolar doses, whereas prior studies of monomeric CPPs typically required micromolar concentrations for detection of internalized proteins [120–122]. Recent work by Tietz et al. [110] further supports the benefits of TAT multimerization for intracellular antibody delivery. In this study, a trimeric cyclic TAT was used to deliver AlexaFluor488-labeled IgG into HeLa cells. Intracellular fluorescence was detected when using 1 μM trimeric cyclic TAT but not with 1 μM monomeric cyclic TAT.

Akin to the method by Mie et al. discussed above, Oh et al. [112] fused an IgG-binding domain to multimeric leucine (L)- and lysine (K)-rich amphipathic α-helical CPPs described earlier. To enable antibody delivery, this platform utilizes Domain Z, an engineered protein derived from IgG-binding protein A. Either LK-2 or LK-4 was genetically fused to the N-terminus of Domain Z to generate non-covalent CPP-fused adaptors for IgG delivery [117]. Simple co-incubation of these LK-Domain Z adapters with mouse IgG2A and human IgG generates non-covalent LK-Domain Z–Antibody complexes with the ability to penetrate cells. While both CPP-Domain Z fusions enabled nanomolar delivery of IgG, LK-4 complexation resulted in higher cellular penetration. Delivery was found to be dose-dependent with respect to CPP-Domain Z up to 200 nM, and complexation between LK-Domain Z was found to be near-instantaneous. Importantly, cellular delivery was highly efficient. Greater than 80% delivery was observed 6 h following incubation with 100 nM IgG complexed with LK-Domain Z. To demonstrate functionality of the LK-Domain Z IgG delivery tool, anti-NFκB antibodies were delivered into hTNF-α-stimulated cells. Abundance of NFκB-dependent transcripts was decreased by up to 50% in cells treated with IgG/LK-Domain Z complexes, whereas free anti-NFκB antibodies had no effect on gene expression.

Cell-penetrating antibodies were generated by Choi et al. [123] by humanizing mouse anti-DNA autoantibodies. These resulting “cytotransmabs” could enter cells via electrostatic interactions of light-chain cationic residues with heparan sulfate proteoglycan (HSPG) membrane receptors resulting in endocytosis. Following binding, cytotransmabs are able to enter the cytosol via pH-induced conformational change and pore formation [124]. This unique format was used to develop a cell-penetrating anti-RAS antibody, RT11 [125]. To improve cytosolic access and therapeutic potency, RT11 was further engineered with an improved endosomal escape motif. This second-generation cell-penetrating anti-RAS antibody, dubbed InRas37, displays ~2-fold greater cytosolic delivery relative to RT11. Crucially, inRas37 demonstrated potent inhibition of various RAS-mutant tumors in vivo when used as a monotherapy or in combination with a PI3K inhibitor [126].

#### CPPs for gene editing

Recently, Zhang et al. [127] investigated combining CPP-fused Cas nucleases with so-called “assist peptides” as an alternative non-viral method of introducing gene editing enzymes into cells. These assist peptides were co-incubated with the CPP/nuclear localization sequence (NLS)-modified Cas9, TAT-4xNLS-Cas9-2xNLS (Cas9-T6N). It was found that co-incubating Cas9-T6N with TAT-HA2 assist peptide potentiated highly efficient gene editing in a panel of primary mouse and human cell lines transduced with viral sgRNA. Notably, HA2 is a known endosomolytic peptide derived from the influenza virus hemagglutinin subunit [128,129]. It adopts a helical conformation in the acidic endosomal environment, promoting fusion with the endosomal bilayer, membrane destabilization, and subsequent release into the cytosol [130]. Interestingly, directly fusing the HA2 peptide to Cas9-T6N did not enhance gene editing. Furthermore, co-incubation of Cas9-T6N with TAT or HA2 alone resulted in negligible gene editing. Of note, the HA2 peptide on its own exhibits poor aqueous solubility, which may contribute to its lackluster performance. These data suggest that TAT-HA2 may be uniquely suited to assist Cas9-T6N cytosolic delivery by complexation in solution rather than genetic fusion. Dubbed peptide-assisted genome editing (PAGE), this method offers rapid (~30 min) and highly efficient (80% to 90%) gene editing in multiple mouse and human primary cells with minimal toxicity.

In a parallel study, Foss et al. [131] developed Peptide-Enabled RNP delivery for CRISPR engineering (PERC). The PERC system also utilizes CPPs/endosomolytic peptides to assist Cas delivery. First, TAT-HA2 and variants were screened for delivery by simple co-incubation of Cas9 RNP with CPPs. Editing efficiency was determined by *B2M* locus by next-generation sequencing (NGS). In good agreement with Cas-PAGE, PERC demonstrated 68% knockout efficiency with just 0.5 μM RNP with A5K, a derivative of HA2-TAT. Although less efficient than electroporation (99% efficiency), PERC consistently resulted in higher viability than electroporation. As with PAGE, PERC successfully edited multiple primary cell types achieving 67% knockout of CD45 in B cells and 17% knockout efficiency of CD45 in NK cells. Beyond Cas-mediated knockout, PERC also enabled base editing and gene knock-in by delivery of either adenine base editors or HDR donors, respectively. However, both of these applications suffered from relatively low efficiencies. For example, PERC co-delivery of RNP and template DNA achieved a 3% knock-in rate compared to 39% by electroporation. In summary, PERC is a versatile Cas delivery method offering efficient ex vivo gene knockout in a variety of primary human cell lines.

Notably, the Cas-PAGE and PERC reports demonstrated TAT-HA2 and A5K respectively could produce similar levels of gene editing in a panel of commercially available Cas9 proteins. Thus, both peptides could potentially enable delivery of unmodified Cas proteins and Cas RNPs, further increasing user convenience, as CPP/NLS-modified Cas9 may be difficult to purify at workable yields.

In another recent example of CPP-mediated Cas delivery, Stahl et al. [132] developed NLS-modified Cas9 for both in vitro gene editing and in vivo mouse brain editing. To achieve intracellular delivery, 4 copies of SV40 NLS were fused to the N-terminus and 2 copies to the C-terminus of Cas9 or SauCas9, generating cell-penetrant 4x-Cas9-2x and 4x-SauCas9-2x. Gene editing in primary mouse neural precursor cells was high at ~60% efficiency with 4x-SauCas9-2x. Editing efficiency of >20% was achieved in human iPSC-derived neural precursor cells following incubation with 4x-Cas9-2x RNPs. Editing of mouse brains was achieved by intracranial infusion of 4x-Cas9-2x. In vivo delivery via cell-penetrating RNPs also resulted in less immune activation compared to AAV delivery, paving the path for safer gene editing strategies for CNS indications.

Taken together, these recent developments in CPP-mediated Cas delivery methods open the possibility of scalable and higher throughput screening of gene and cell therapies. By relying on CPPs, these methods reduce the hazards and cytotoxicity associated with viral vectors and electroporation respectively. Especially for Cas-PAGE and PERC, addition of TAT-HA2 or A5K assist peptides significantly improves Cas delivery relative to older CPP-mediated Cas delivery methods suffering from low delivery/editing rates in primary cells [133] and/or cumbersome conjugation requirements [134].

#### CPPs for targeted protein degradation

**Fig. 2.**
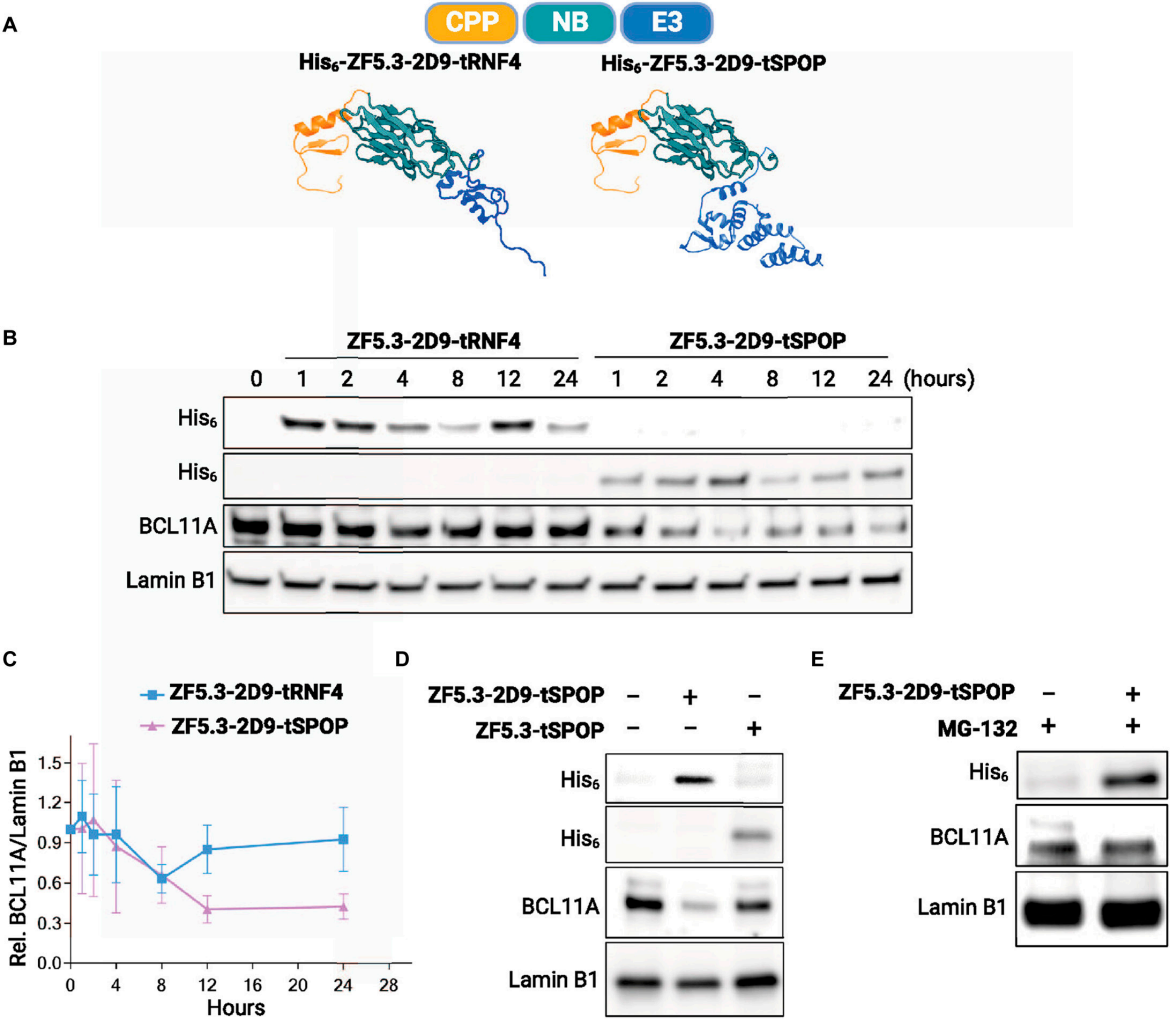
Harnessing CPPs for bioPROTAC delivery and undruggable target degradation. (A) Design of BCL11A-targeting bioPROTACs fused to a helical ZF5.3 cationic CPP. (B) Western blots demonstrate intracellular uptake of bioPROTACs and reveal ZF5.3-2D9-tSPOP as an active BCL11A degrader. (C) Time course of BCL11A degradation in HUDEP-2 cells when treated with 10 μM CPP-fused bioPROTACs. (D) Degradation is nanobody (2D9)-dependent. (E) The proteasome inhibitor MG-132 rescues BCL11A degradation, validating the bioPROTAC mechanism of action. This figure was reproduced under a CC BY 4.0 license and is attributed to Shen et al. [135].

Recently, Shen et al. [135] developed a CPP-based method that was employed to deliver BCL11A-degrading bioPROTACs cytosolically. The BCL11A transcription factor is a negative regulator of fetal hemoglobin (HbF). Increasing HbF levels via BCL11A knockdown is seen as one possible therapeutic avenue for the treatment of sickle cell disease and β-thalassemia, but BCL11A remains an undruggable target. To target BCL11A, Shen et al. first identified a BCL11A-specific nanobody using yeast display. The optimized 2D9 nanobody was subsequently fused to both the ZF5.3 CPP and an E3 ligase domain, tSPOP, to generate a cell-permeable BCL11A degrader (Fig. 2). Incubation of human umbilical cord blood-derived erythroid progenitor (HUDEP)-2 cells with 10μM of ZF5.3-2D9-tSPOP led to a 70% reduction in endogenous BCL11A within 12 h. Moreover, ZF5.3-2D9-tSPOP treatment of HUDEP-2 cells and primary CD34+ progenitor cells induced significant HbF production at 4 and 9 days post-treatment, respectively. Excitingly, this bioPROTAC could spare the closely related BCL11B paralog, highlighting bioPROTACs as highly specific molecular tools for targeted degradation.

#### Bacterial toxins

Just as CPPs originated from viral proteins, bacteria have also evolved machinery to deliver virulence factors into host cells. Bacterial toxins commonly investigated for protein delivery include Diphtheria toxin, Anthrax toxin, and Pseudomonas exotoxin A [136]. These toxins operate as 2-component “AB” systems. The binding of the “B” moiety to cell surface receptors mediates delivery of the effector “A” domain. In Diphtheria exotoxin, endosomal escape is afforded by an additional translocation domain, while the B moiety of Anthrax toxin forms heptameric pre-pores allowing protein escape from endosomes into the cytosol. Considerable effort has been made to repurpose these naturally penetrant toxins into protein delivery platforms. Importantly, non-toxic variants of these bacterial proteins have been engineered for protein delivery without cytotoxicity [137,138].

Early work by Olsnes and colleagues [139,140] showed that it was possible to fuse cargo proteins to the complete Diphtheria toxin N-terminus in order to piggyback off of AB toxin endocytosis. Diphtheria toxin-mediated intracellular delivery was further expanded to include diverse protein payloads such as folded fluorescent proteins as well as functional enzymes by the Melnyk group [141]. Using this method, the Melnyk group delivered functional purine nucleoside phosphorylase into patient-derived cells as an enzyme replacement strategy [137].

Unlike Diphtheria and Pseudomonas toxins, Anthrax toxin comprises 2 discrete proteins. The receptor-binding protective antigen (PA) aids cytosolic delivery of the effector proteins: lethal factor (LF) or edema factor (EF). After EF or LF binds PA, the entire complex enters cells via endocytosis, and the virulence factors escape into the cytosol via pores formed in acidic late endosomes [136]. In typical applications harnessing Anthrax toxin, the protein payload is fused to either the whole LF protein [142] or the non-toxic N-terminal fragment of LF (LF_N_) [143]. The LF/LF_N_-cargo fusion is then combined with WT PA or engineered PA with modified receptor specificity [144]. In a notable study, Mechaly et al. [145] reprogrammed Anthrax toxin specificity by first mutating 2 residues on PA essential for native receptor recognition and then fusing EGF to the binding-deficient PA to generate EGFR-targeting PA. Co-incubation with both the engineered PA and an LF-fused Diphtheria toxin caused rapid cell death in A431 lung cancer cells expressing high levels of EGFR but spared CHO-K1, a cell line that does not express the receptor. Further validating the entry mechanism, adding free EGF to culture media prevented cell death by competing with EGFR on cell surfaces. With this method, LF-fused proteins can also be successfully redirected to HER2+ breast cancer cells by replacing EGF with an anti-HER2 affibody [146] and to epithelial cell adhesion molecule (EpCAM)-expressing cells with a high-affinity DARPin [147,148]. Beyond toxins, other proteins successfully delivered by PA/LF_N_ include affibodies, monobodies, and DARPins [138].

### Nanocarrier-mediated protein delivery

In contrast to direct protein engineering methods, protein delivery by nanoscale carriers (1 to 1,000 nm) relies on “vehicles” to shuttle protein payloads across cell membranes. Whether encapsulated within nanoparticle cores, complexed as a macromolecular assembly, or tethered to the nanostructure surface, nanocarriers afford high loading capacity and enhanced membrane penetration. In addition, nanomaterials shield proteins from enzymatic degradation, can decrease immunogenic response compared to free proteins, and can facilitate tissue-specific targeting either by intrinsic material properties or by surface-conjugated ligands.

Several classes of nanocarrier methods are discussed in this section. Liposomes and more advanced LNPs are surveyed. In parallel with lipids, polymers have also been developed for protein delivery. Advancements in polymer chemistry have resulted in a rich collection of materials with an enormous assortment of physical properties such as charge, hydrophilicity/hydrophobicity, and functional groups. Owing to their versatility and customizability, polymers have seen intense interest as protein nanocarriers and are typically formulated as micelles or supramolecular complexes [149]. Inorganic particles based on biocompatible gold or silica have also been extensively investigated for protein delivery [150,151]. Finally, bioinspired nanocarriers including virus-like particles (VLPs) and cell-derived exosomes are introduced.

#### Liposomes

Some of the simplest lipid-based nanoparticles are liposomes, vesicular nanoparticles with aqueous cores enclosed by phospholipid membranes. Unilamellar or multilamellar liposomes can be easily generated by techniques such as thin-film hydration, reverse-phase evaporation, and solvent injection [152]. Protein cargo, being hydrophilic, are typically entrapped within the aqueous compartment as demonstrated by early attempts at loading transcription factors [153], lysozyme [154], and horseradish peroxidase [155] into liposomes. Unfortunately, classical liposomes generated by thin-film hydration suffer from low protein loading efficiency [156] as well as poor cellular uptake and/or endosomal escape due to their inert surfaces. Protein encapsulation with liposomes can be improved from single-digit efficiencies to >40% with repeated freeze–thaw cycles [156,157]. To address poor uptake and endosomal escape, fusogenic lipids such as 1,2-dioleoyl-sn-glycero-3-phosphoethanolamine (DOPE) can be incorporated into liposomal formulations to destabilize endosomal membranes and increase cytosolic access [158]. In some cases, liposomes incorporating both DOPE and a cationic lipid can fuse with cell membranes, bypassing endocytosis altogether for direct cytosol delivery [159,160].

Ultimately, because proteins vary so widely in size, conformation, and surface charge, generalizable methods for LNP encapsulation of unmodified proteins are challenging. Instead, research groups have turned to co-engineering both the protein payloads and lipid carrier, improving interactions through enforced physiochemical properties. For example, Kim et al. [161] conjugated membrane-permeable peptides to cargo protein. The modified proteins could associate with pre-formed liposomes by insertion into the membrane bilayer in stark contrast to previous methods relying on entrapment within aqueous cores. This alternate approach enabled high loading efficiency (60% to 70%) and rapid cytosolic delivery of cargo protein. Delivery of pro-apoptotic cytochrome *c* by this strategy induced H460 lung cell death in a mouse xenograft model.

#### Lipid nanoparticles

Unlike liposomes, LNPs have a single lipid outer layer, are tightly packed with lipids, and lack vesicular structure. LNPs can also self-assemble by vigorous pipette mixing or microfluidic mixing under milder solvent conditions relative to liposomes making them attractive for encapsulation of fragile bioactive cargo.

Charge modification is one prominent method to enable lipid encapsulation of protein cargo by augmenting electrostatic interactions between lipid and proteins. This method aims to endow proteins with a net negative charge similar to nucleic acids. Cationic or ionizable lipids and lipidoids typically used for nucleic acid transfection may then be used as delivery vehicles for these negatively charged proteins. With the advancement of gene therapy technologies, considerable strides have been made toward LNP-mediated delivery of anionic siRNA, mRNA, and DNA. Pioneering work on combinatorial lipid synthesis introduced methods to rapidly generate vast libraries of structurally diverse lipids for RNA encapsulation and delivery [162,163]. For LNP-based transfection, both siRNA-mediated knockdown and mRNA gene expression are highly dependent on lipid properties [164], suggesting the need for lipid screening in protein delivery as well. Thus, cytosolic protein delivery can be built on this wealth of available lipids, and these same lipids developed for nucleic acid delivery may be co-opted for development of LNPs carrying charge-modified protein cargo.

In one of the first demonstrations of protein charge engineering for LNP delivery, Wang et al. [165] reacted cargo protein with excess cis-aconitic anhydride, converting positively charged lysine groups into negatively charged carboxylate groups. Chemically modified cytotoxic proteins including RNase A and saporin could then be encapsulated by cationic LNPs for in vivo tumor inhibition. In an alternate approach, the Liu group demonstrated cationic lipid-mediated delivery of gene editing enzymes by fusion with supernegative GFP [166] or other naturally derived anionic proteins [167]. To demonstrate proof of concept, charged fusions of Cre recombinase were complexed with commercially available cationic lipids such as Lipofectamine for cytosolic delivery. Importantly, protein function was not compromised by these additional charges. In fact, previous work had demonstrated enhanced stability of some proteins via supercharging [168].

Beyond genetic and chemical charge modification, nucleic acids may also be used to impart anionic properties onto cargo proteins. For example, Eltoukhy et al. [169] conjugated DNA oligos to proteins to increase negative charge density and enable LNP encapsulation. This finding is made more salient with the rise of CRISPR/Cas gene editing tools, as Cas enzymes naturally associate with guide RNA. In this case, pre-loading Cas enzymes with sgRNA confers sufficient negative charge to incorporate the entire Cas:sgRNA complex into ionizable LNPs. Here, the RNA serves dual functions: gene targeting and facilitation of LNP encapsulation without the need for further protein modification. In proof-of-concept studies, Cas9 RNPs were either complexed with Lipofectamine or encapsulated by LNPs for subsequent cytosolic delivery and gene editing [166,170,171]. Notably, there is a strong structure–function relationship for ionizable cationic lipid delivery of RNPs. In a survey of combinatorial ionizable lipids for RNP delivery, out of 12 lipids tested, 5 lipids enabled >50% GFP knockdown when encapsulating GFP-targeting Cas RNPs [170]. Moreover, up to 70% knockdown was achieved in 3 of the lipids, whereas no gene editing was observed with treatment of RNPs alone.

To date, multiple reports of RNP delivery by ionizable/cationic LNPs have been published. In one study, Cheng et al. [172] showed that LNPs encapsulating RNP complexes could be delivered to the spleen, liver, and lungs in a reporter mouse strain as determined by tdTomato expression following deletion of an upstream stop cassette. The group also targeted phosphatase and tensin homolog, a known cancer suppressor, with LNP-delivered RNPs, achieving up to 5% editing efficiency of the endogenous gene in vivo. In a parallel study, Wei et al. [173] systematically examined RNP loading into LNPs and dissected key parameters for high loading capacity and efficient intracellular delivery. It was found that adding 1,2-dioleoyl-3-trimethylammonium-propane (DOTAP), a cationic lipid, to ionizable LNP formulations facilitated RNP encapsulation under neutral pH conditions. Optimal gene editing was achieved when 10% to 20% molar percentage of DOTAP was incorporated into LNP formulations owing to increased electrostatic complexation with negatively charged RNPs. Gene editing of other organs including the eye have also been demonstrated via local injection of lipid:RNP complexes [174,175].

**Fig. 3.**
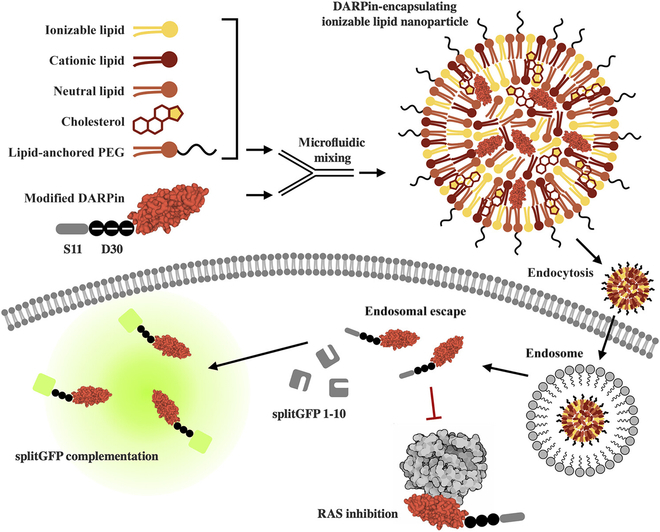
LNPs for the intracellular delivery of ApP-fused protein cargo. Therapeutic DARPins are fused with a negatively charged ApP sequence as well as a split GFP S11 tag. The protein cargo is encapsulated within LNPs via microfluid mixing with lipids, cholesterol, and polyethylene glycol (PEG). Formulated LNPs efficiently transport protein cargo across cell membranes, facilitate endosomal escape, and enable specific inhibition of undruggable targets. Cytosolic delivery can be detected with a split GFP complementation assay in reporter cells expressing the GFP(1–10) fragment. Reprinted (adapted) with permission from Haley et al. [179]. Copyright 2023 American Chemical Society.

**Fig. 4.**
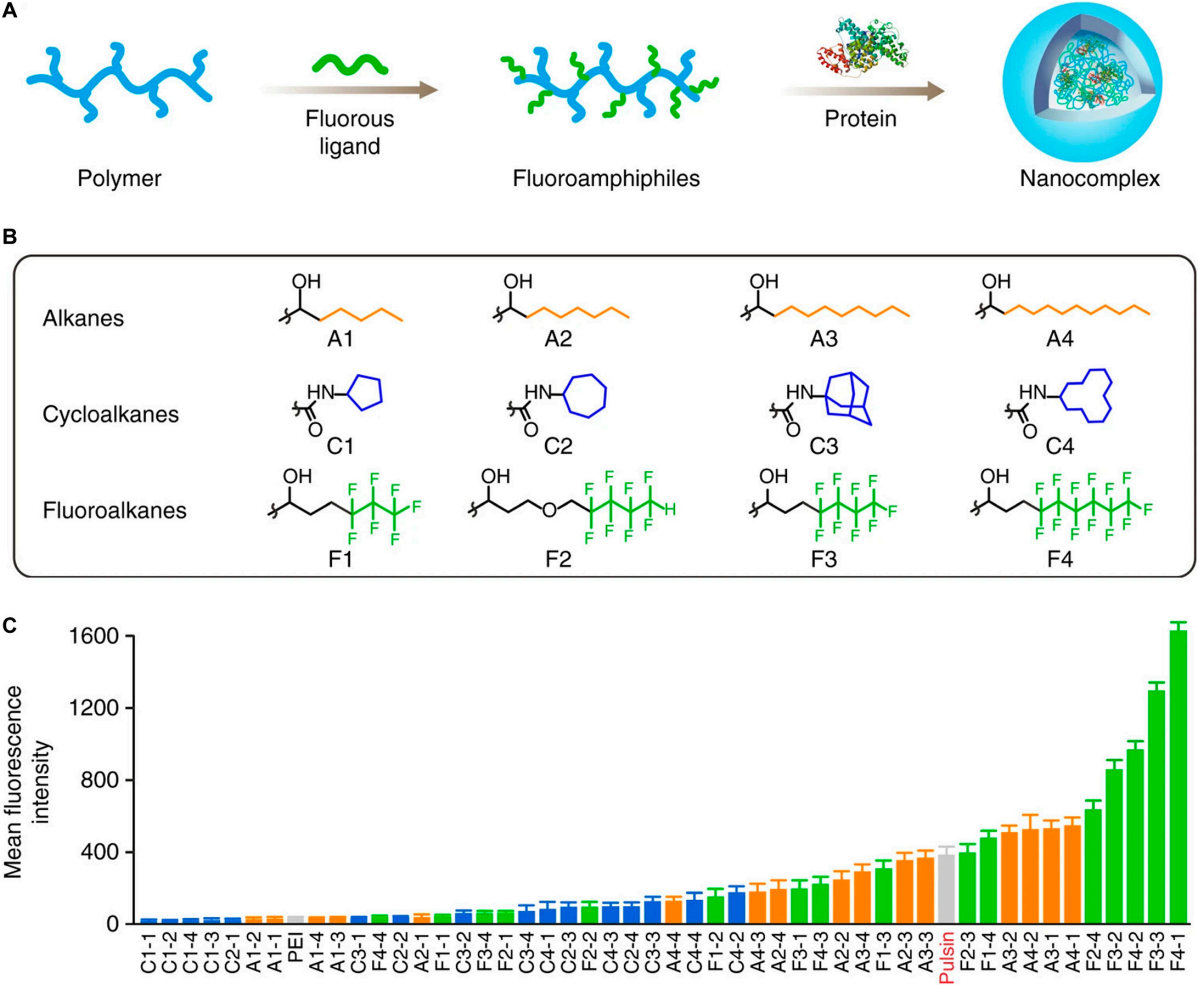
Fluoroalkyl-grafted polymers demonstrate enhanced protein uptake. (A) Schematic for grafting functional chains onto branched PEI for protein co-assembly. (B) Structures of alkanes (A1 to A4), cycloalkanes (C1 to C4), and fluoroalkanes (F1 to F4) grafted onto PEI polymer backbones. (C) Intracellular delivery of FITC-labeled BSA with a library of conjugated PEI polymers and controls. The number after each dash represents the degree of PEI functionalization with 1 being the lowest and 4 being the highest. This figure was reproduced under a CC BY license and is attributed to Zhang et al. [197].

While methods for lipid-mediated delivery of RNPs are now well-established, Cas enzymes represent a special case as LNP cargo. Most therapeutically interesting proteins cannot bind nucleic acids for cationic lipid complexation or LNP encapsulation and require charge conjugation by chemical or genetic means. To address this limitation, our group developed a universal method for IgG delivery by fusing photoreactive antibody binding domains (pAbBD) to a minimal anionic polypeptide (ApP). These antibody adaptors are engineered with both a non-canonical amino acid, benzoyl-phenylalanine, for light-activated crosslinking [176] and C-terminal polyaspartic acid or polyglutamic acid ApPs [177]. Covalent attachment of negatively charged ApPs to IgGs via pAbBDs allowed us to complex antibodies with Lipofectamine for efficient cytosolic delivery. We further demonstrated that ApPs enabled efficient lipid-mediated delivery of commonly used small-protein scaffolds including DARPins and nanobodies [178] (Fig. 3). By tuning key parameters such as buffer pH, ionizable lipid structure, excipient amounts, and lipid-to-cargo ratio, an LNP formulation was developed achieving ~70% DARPin encapsulation. This optimized LNP formulation could be used for in vivo delivery of anti-RAS DARPinK27 (Fig. 4) to mouse livers as a cancer therapeutic [179]. Importantly, we and others have shown that controlled charge modification of cargo proteins does not affect binding affinity [178], solubility, or protein folding [170].Thus, this method enables loading of proteins into LNPs under mild synthesis conditions for efficient in vitro and in vivo cytosolic delivery.

#### Charged polymers

As with many LNP methods, polymer–protein interactions are dramatically improved by engineering charged groups onto cargo proteins. This can be achieved by either chemical conjugation of anionic chemical moieties such as citraconic anhydride or genetic fusion to negatively charge peptides or protein domains. In one example, diblock copolymers containing a cationic portion were able to form stable micelles with charge-modified proteins [180]. In another method, polymers were grafted with guanidinium groups to generate positively charged materials for protein complexation [181,182]. Interestingly, similar guanidinium-rich polymers have been reported for delivery of unmodified, native proteins [183,184], although recent work suggests that negative charge association is indeed essential for high-efficiency complexation and internalization [185]. Crosslinked cationic polymers have been reported to stabilize proteins within nanoparticles, offering a method to combat formulation issues arising from weakly charged cargo [186].

Cationic, anionic, and zwitterionic versions of poly(β-amino ester) (PBAE) have also been developed for delivery of a variety of protein payloads including IgGs, GFP, saporin, and bovine serum albumin (BSA) [187,188]. To demonstrate potential clinical applications of PBAE-delivered proteins, Rui et al. [188] encapsulated RNPs into multifunctional, carboxylated PBAE polymer nanoparticles and demonstrated efficient GFP knockdown in cell cultures and in mouse brains following intracranial injection. Notably, net neutral PBAEs were also shown to complex with diverse native protein cargo, suggesting that alternate factors beyond electrostatic interactions are also critical for polymer-mediated protein delivery. Specifically, carboxylate groups [188] and phosphocholine groups [187] grafted onto polymers are able to form hydrogen bond networks with the functional groups of protein surface residues.

Charged polymers have been utilized for cancer vaccination approaches as well. In one example, Foster et al. [189] conjugated ovalbumin to anionic poly(propylacrylic acid) and complexed the resulting protein–polymer molecule with cationic poly(dimethylaminoethyl methacrylate) to produce ovalbumin–polymer particles. These ovalbumin–polymer complexes enhanced cytosolic accumulation in macrophages in vitro. In addition, vaccination with these ovalbumin nanoparticles prior to a challenge with ovalbumin-expressing cancer cells protected mice from tumor development. Cationic polymer brushes [190] and cationic micelles [191] have also been reported for ovalbumin loading and APC activation.

#### Coordinative polymers

Polymers have also been engineered with coordination groups for association with affinity-tagged proteins. These methods are based off of coordination chemistry principles more commonly used for metal-affinity protein purification. Postupalenko et al. [192] synthesized an anionic polymeric backbone laden with nitrilotriacetic acid immobilized nickel (Ni-NTA) groups. The nickel ions could capture proteins containing the common 6-His affinity tag to generate protein-bound polymer. Finally, a cationic polyethylenimine (PEI) polymer was used to encase the entire anionic polymer:protein strand to form nanocomplexes for cytosolic delivery. Ren et al. [193] produced polymeric dendrimers surface-decorated with dipicolylamine-zinc ion coordination complexes. Here, the zinc ion could bind cargo protein via protein imidazole and amine groups as well as through π–π interactions. These zinc coordination dendrimers were successfully utilized for delivery of a diverse panel of proteins into multiple cell lines. A dose-dependent decrease in cell viability was observed with delivery of cytotoxic proteins: saporin, RNase A, and α-chymotrypsin, indicating bioactivity following delivery. Recently, native protein delivery was also demonstrated by Zn^2+^, Ni^2+^, and Cu^2+^ immobilized dendrimers [194].

#### Boronic acid polymers

Boronic acid has emerged as a promising functional group for native protein binding. The electron-withdrawing boronic acid group enables binding to both cationic and anionic residues via electrostatic, nitrogen–boron, and cation–π interactions. Liu et al. [195] exploited these diverse binding modalities of boronic acid and developed cationic dendrimers enriched with phenyl boronic acid for native protein complexation and cytosolic delivery. These polymers could complex and deliver proteins spanning molecular weights from 12 kDa to 430 kDa and isoelectric points from 4 to 11. Notably, fluorescent proteins, enzymes, toxins, and Cas were delivered by boronic acid dendrimers and retained proper folding and functionality following cytosolic delivery. Protein delivery was demonstrated at concentrations on the order of ~10 nM. Recently, boronic acid-rich cationic polymers were utilized for in vivo delivery of saporin for the treatment of a mouse osteosarcoma model [196].

#### Fluorinated polymers

Fluorous groups have also demonstrated promising protein coupling effects. Zhang et al. [197] grafted fluoroalkyl moieties (alkyl chains with fluorine atoms substituting hydrogen atoms) to branched PEI polymers. These fluorinated polymers were able to form uniform nanoparticles with proteins by co-incubation. This fluorination effect allowed encapsulation and delivery of native proteins at 300 nM protein dose. Cells internalized fluorinated polymer nanoparticles via endocytosis, and fluorescein isothiocyanate (FITC)-labeled proteins could efficiently escape endosomes as confirmed by disperse cytosolic fluorescence (Fig. 4). The Cheng group has developed several fluorous polymers that readily complex with cargo protein to produce stable nanoparticles [198,199]. As with other studies, saporin was used as a model cargo protein for in vivo delivery via fluoropolymer nanoparticles for successful tumor growth inhibition. Fluorous amphiphiles are thought to stabilize cargo protein primarily via hydrogen bonding [200], but the exact mechanisms driving fluoropolymer-mediated protein encapsulation and cytosolic delivery are still poorly understood.

#### Gold nanoparticles

Gold nanoparticles (AuNPs) have been widely developed as protein delivery vehicles owing to their low toxicity. Protein delivery by AuNPs invariably relies on surface functionalization of binding ligands to enable adsorption or conjugation of cargo proteins. Surface functionalization of AuNPs is often achieved through self-assembly of thiol-containing ligands via strong gold–sulfur bonds. Ghosh et al. [201] modified AuNP surfaces with short cationic peptides (HKRK) to interact with intrinsically anionic β-galactosidase. The β-galactosidase proteins adsorbed onto AuNP surfaces and could cross cell membranes, escape the endosome, and retain enzymatic activity. Protein transfection efficiency was determined by X-gal hydrolysis, and a delivery efficiency of >80% was determined following treatment with 50 nM β-galactosidase. To extend this method for delivery of proteins with diverse isoelectric points, an anionic peptide tag comprising glutamic acid repeats can be fused to cargo, thereby enforcing a negative charge. Anionic proteins can electrostatically couple with arginine-functionalized AuNPs (2 nm) to form hierarchal AuNP:protein gold nanoclusters (>100 nm). These protein gold nanoclusters fuse with the cell membrane to deliver cargo protein [202]. Electrostatically assembled gold nanoclusters were later extended for gene editing applications [202,203]. Using this approach, RNP AuNP clusters were delivered in vivo by intravenous injection for editing efficiencies of 4% and 8% in hepatic and splenic macrophages, respectively [204].

For Cas delivery, DNA can also be used to template RNPs onto AuNP surfaces. Wu et al. [205] designed AuNPs functionalized with photolabile DNA strands complementary to Cas-bound guide RNA to form an RNP corona for spatiotemporally controllable gene editing. Lee et al. [206] hybridized donor DNA to AuNP surfaces via a thiol-oligo and complexed RNPs to the resulting DNA-templated AuNPs. The entire complex was coated with cationic polymers that enhance cellular uptake. With these complex DNA-assembled AuNP:RNP particles, simultaneous delivery of RNP and donor DNA enabled homology-directed repair with a single all-in-one delivery system. DNA-assembled AuNPs also display dramatic increases to hydrodynamic size potentially indicating AuNP clustering as with peptide-assembled RNP AuNPs. In a recent study, gold nanorods were coated with enhanced green fluorescent protein (EGFP) and RNase A as a pre-formed biomimetic protein corona [207]. RNA degradation was demonstrated following delivery of AuNP coated with RNase A in cancer cell spheroids.

#### Silica nanoparticles

Biocompatible silica/silicon dioxide nanoparticles (SiNPs) have also emerged as a popular inorganic vehicle to shuttle proteins into cells. Bale et al. functionalized 20-nm SiNPs with hydrophobic n-octadecyltrimethoxysilane (n-ODMS). These functionalized SiNPs could adsorb proteins onto its surface including BSA and IgGs. Functionality of adsorbed proteins was demonstrated by delivery of an inhibitory anti phospho-Akt antibody into MCF-7 breast cancer cells resulting in apoptosis. Niu et al. [208] fabricated n-ODMS-treated rough SiNPs resembling viruses in morphology by coating large core particles with smaller shell particles. Importantly, the bumpy topology had an additive effect on top of hydrophobic coating in aiding protein entrapment [209].

In contrast to solid SiNPs, mesoporous SiNPs are hollow and can trap proteins throughout their tortuous channels [210]. Methods to precisely tune porosity, pore size, and wall thickness of mesoporous SiNPs make these particles amenable to a variety of protein cargo including cytochrome *c* [210], RNase A [211], IgG [212], and ovalbumin [213]. Owing to their high surface area, mesoporous SiNPs exhibit high protein loading capacities with recent reports indicating loading capacities up to 40% protein/SiNP mass [214].

#### Virus-like particles

Inspired by the broad tropism and infectivity of viruses, researchers have sought to generate VLPs for protein delivery by fusing cargo proteins to retroviral genes. Following transfection of fusion genes into eukaryotic host cells, viral components including cargo-fused capsid/matrix proteins self-assemble into VLPs. In one of the first studies demonstrating the feasibility of viruses for protein delivery, Voelkel et al. [215] cloned cargo proteins into the retroviral Gag-Pol gene to package proteins into virions. Later, Kaczmarczyk et al. [216] improved the safety of VLPs by removing the Pol gene altogether to generate replication-deficient VLPs, since Gag alone is the minimal unit necessary for viral assembly. Protein delivery by VLPs has been demonstrated for a variety of proteins including recombinases [215,216] and fluorescent proteins [216]. Proteins delivered by VLPs retained functionality following encapsidation into viral particles. Unsurprisingly, VLPs have also been utilized for Cas9 delivery and gene editing applications in recent years. Initial versions of Cas9 VLPs offered modest editing efficiencies in both immortalized and primary human cells [217,218]. In a recent report, Banskota et al. [219] redesigned the Gag-cargo fusion protein and tuned host-cell transfection protocols to improve VLP efficiency. To increase Cas9 and base editor encapsulation within VLPs, a nuclear export sequence was incorporated into the Gag-Cas fusion protein to facilitate packaging of gene editing enzymes. Additionally, the ratio of cargo-to-packaging plasmids transfected into production cells was optimized. As a result, base editor packaging was improved by 11-fold, and editing efficiencies increased by more than 5-fold relative to the original VLP design.

While many methods for VLP production discussed here rely on expression of viral components in eukaryotic host cells, methods for in vitro VLP assembly of *E. coli* purified capsomere–cargo fusions have also been reported [220].

#### Exosomes

Exosomes are vesicles secreted by cells having a myriad of reported biological functions from long-distance communication to promoting immunity [221]. These functions are mediated by cell-to-cell delivery of nucleic acids, metabolites, and proteins loaded into vesicles during exosome formation. With high serum stability and a natural propensity for crossing cellular membranes, exosomes have been repurposed to load exogenous proteins for cytosolic delivery.

The most common methods for loading therapeutic molecules into exosomes are based on physical disruption of the lipid bilayer. Techniques like electroporation, sonication, and freeze–thaw cycles have been applied to load diverse protein cargo into isolated exosomes [222]. Wan et al. used electroporation to load RNPs into exosomes harvested from hepatic stellate cells for liver gene editing. The majority of exosomes were taken up by hepatocytes via endocytosis enabling treatment of multiple disorders including acute liver injury, fibrosis, and liver cancer [223].

**Fig. 5.**
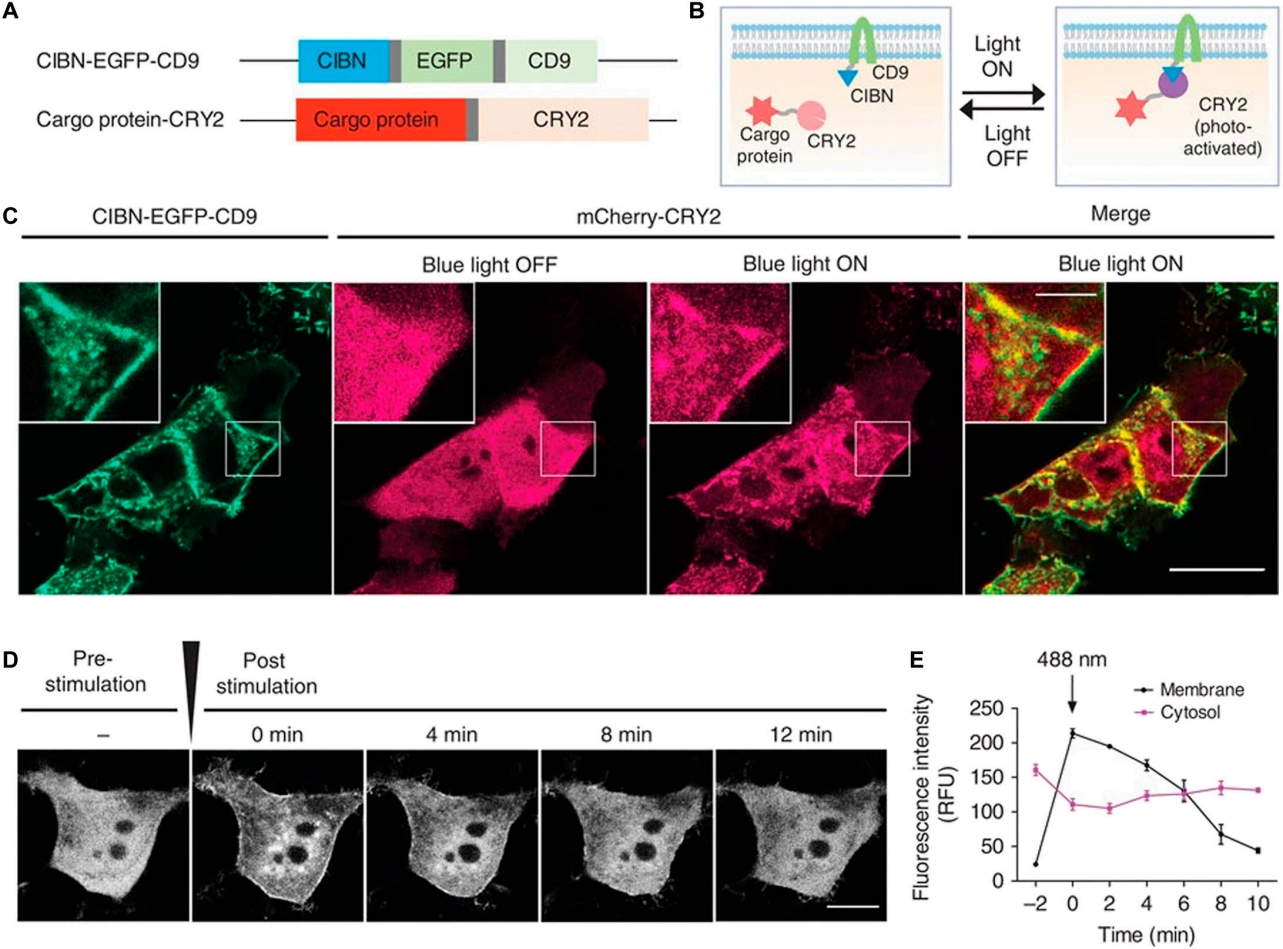
EXPLORs for coupling exosome cargo loading and biogenesis. (A) Fusion protein constructs used for optogenetic recruitment of cargo to exosomes. (B) Blue light illumination induces recruitment of mCherry-CRY2 to membrane-bound CIBN-CD9. (C) Representative fluorescent images of HEK293T cells expressing EXPLOR components with or without 488-nm laser stimulation. Cargo co-localizes to the inner leaflet upon illumination. Scale bars are 20 μm and 5 μm for whole and inset images, respectively. (D) Time course images of mCherry-CRY2 signal in cells pulsed with blue light. (E) Quantitation of mCherry-CRY2 signal in both cytosolic and membrane subcellular compartments following blue light stimulation. This figure was reproduced under a CC BY license and is attributed to Yim et al. [224].

**Fig. 6.**
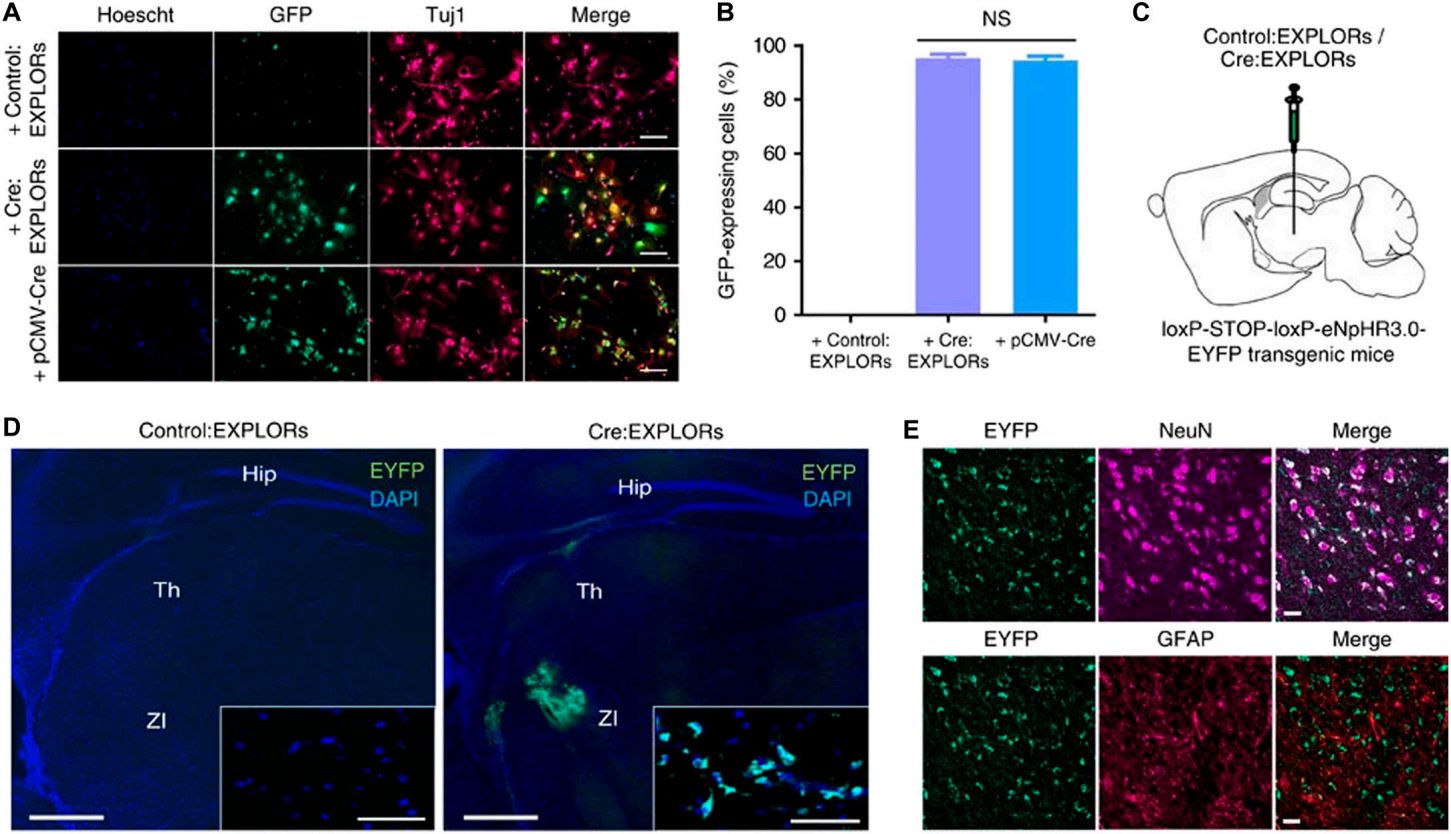
In vivo delivery of exosomes manufactured by the EXPLORs method. (A) Differentiated neurosphere-derived cells expressing loxP-STOP-loxP-EGFP were treated with Cre:EXPLORs or transfected with pCMV-Cre vectors. Fixed cells were stained with antibodies against a neuron-specific class III beta-tubulin marker, Tuj1, GFP, and Hoechst 33342. Scale bars are 100 μm. (B) Percentage of EGFP-expressing cells following treatment. (C) Schematic for the administration of Cre:EXPLORs in loxp-stop-loxp-eNpHR3.0-EYFP transgenic mice by ventrolateral injection. (D) Fluorescent images of fixed brain slices following EXPLOR injection in transgenic mice. Green fluorescence indicates eNpHR3.0-EYFP protein expression and blue fluorescence indicates cell nuclei. Insets show high-magnification detail in neurons. Scale bars are 500 μm and 50 μm for whole and inset images, respectively. Hip, hippocampus; Th, thalamus. (E) Representative image of NeuN/GFAP immunohistochemistry of the brain following Cre:EXPLORs treatment. Recombination occurs mainly in (NeuN)-positive neurons rather than red, glial fibrillary acidic protein (GFAP)-positive astrocyte cells. Scale bars are 20 μm. This figure was reproduced under a CC BY license and is attributed to Yim et al. [224].

In a synthetic biology approach, Yim et al. [224] developed EXPLORs (exosomes for protein loading via optically reversible protein–protein interactions) for directed protein loading into exosomes within cells. This method requires cells to express 2 fusion proteins: a cargo-cryptochrome 2 (CRY2) fusion and a CIB1 fragment (CIBN) fused to the exosome-associated transmembrane protein, CD9. Blue light illumination of engineered cells induces CRY2 binding to CIBN-CD9 and subsequent loading into exosomes during biogenesis (Fig. 5). Following exosome loading, the cargo protein is released from vesicular membranes when the light source is removed. Exosomes harvested by the EXPLOR method demonstrated loading of various cargo including fluorescent proteins and NF-κB inhibitors. Exosomes loaded with Cre recombinase demonstrated in vivo cytosolic delivery in mouse neurons expressing EYFP downstream of a floxed stop codon (Fig. 6).

To establish the clinical applicability of EXPLORs, super-repressor IκB (srIκB) protein was loaded into exosomes to suppress NF-κB-mediated inflammatory signaling in a number of diseases. When systemically administered in a mouse model of sepsis, srIκB-loaded exosomes were primarily delivered to neutrophils and macrophages. Exosome-mediated inhibition of NF-κB signaling decreased inflammation and increased survival in mice [225]. Kim et al. [226] treated post-ischemic mice with srIκB-loaded exosomes to suppress the NF-κB signaling cascade activated during kidney ischemia–reperfusion injury. Recently, these srIκB-loaded exosomes were also employed to suppress inflammation in models of alcohol-associated liver disease [227] and neuropathic pain [228].

### Emerging protein delivery methods

In addition to physical methods, direct protein engineering, and nanocarrier-mediated delivery, entirely new approaches to intracellular protein delivery methods are also being explored. In a recent example, improved understanding of native biological transport systems precipitated a cytosolic protein delivery tool whereby bacterial syringe-like nanostructures were harnessed to inject recombinant proteins into mammalian cells [229]. In this study, an extracellular contractile injection system (eCIS) derived from the entomopathogenic bacterium *P. asymbiotica* virulence cassette (PVC) was engineered for non-native cytosolic protein delivery. These engineered PVCs demonstrated delivery of Cas9 and zinc finger deaminases for genome engineering when reprogrammed with the human cell targeting Ad5-knob protein. In addition, near-complete killing of A549 and A431 cancer cells was achieved by retargeting toxin-loaded PVCs with an anti-EGFR DARPin. Finally, PVCs installed with anti-mouse binders were shown to deliver proteins to neurons in live animals. By engineering a natural bacterial spike complex, this work introduces a new intracellular protein delivery tool that operates unlike traditional methods previously explored.

A major obstacle for many CPP and nanoparticle systems is endosomal entrapment of protein cargo. Insufficient endosomal escape leads to poor therapeutic performance, often despite high transfection efficiency, and the excessively high doses needed to produce biological effects could trigger off-target toxicities. New chemical delivery tags that do not rely on charge or endosomal uptake mechanisms could provide a means to improve cytosolic delivery, reduce drug dosing, and mitigate potential side effects. In one such approach, Tai et al. [230] chemically modified Coomassie blue with cholesterol to create universal protein delivery tags. Protein cargo can be non-covalently masked with these cholesterol tags to bypass endosomal uptake, directly cross the lipid bilayer, and efficiently enter the cytosol. This work demonstrated efficient cytosolic delivery of various proteins in a broad range of primary and immortalized cancer cell lines. As a proof-of-concept cancer therapeutic, cytochrome *c* was delivered into HeLa cells via these cholesterol tags and demonstrated cytotoxicity, whereas cytochrome *c* alone did not lead to cell death.

## Intracellular Protein Delivery: Challenges and Perspectives

While many advancements have been made toward cytosolic delivery of proteins, none of the discussed technologies have progressed into marketed therapeutics. Here, we highlight some of the critical obstacles facing each method of intracellular delivery as well as for the field as whole. In addition, we offer a perspective on future technological advancements.

### Physical delivery methods

Despite applications in ex vivo cell engineering for adoptive cell transfer, physical protein delivery methods are generally not useful as a therapeutic modality. Microinjection is a low-throughput technique for single-cell delivery, and while electroporation can transfect proteins in bulk, it is normally reserved for ex vivo cell engineering via delivery of Cas RNPs. Thus, other CPP and nanocarrier-based delivery strategies have been the primary focus of many researchers developing cytosolically available protein-based drugs.

One area where physical protein delivery can provide a distinct advantage is in the development of novel intracellular protein drugs, since membrane deformation is an efficient and universal strategy for inducing protein uptake. This allows researchers to quickly validate native protein behavior upon entry to the cytosol without the need for additional protein or nanoparticle engineering. Native protein delivery via physical methods can accelerate mechanistic interrogation and reduce confounding biological outcomes induced by protein modifications or exogenous nanomaterials. Recently, these benefits were highlighted with the development of TRIM-Away [73], a generalized method for targeted protein degradation. Proof-of-concept TRIM-Away studies relied on microinjection and electroporation for cytosolic antibody delivery to demonstrate this powerful and modular method for protein degradation. Later, Sui et al. [231] developed a polymer nanogel-mediated antibody delivery method, opening the possibility of translating TRIM-Away from a research tool into a potential targeted degradation drug.

### Direct protein engineering methods

A major benefit of protein delivery exploiting CPPs or bacterial transduction domains is simplicity. Protein drugs fused or conjugated with CPPs can be administered following purification without a second encapsulation/complexation step as necessitated by nanocarrier methods. Without the need for additional polymer or lipid synthesis, manufacturing costs and development time for direct protein delivery systems are low. For ex vivo applications, including adoptive cell therapy, this reduction in complexity can dramatically improve production throughput.

In general, however, CPPs perform poorly and are prone to endosomal entrapment [232]. Advancements such as cyclic CPPs and multimeric CPPs have improved delivery compared to first-generation linear CPPs, but many of these peptides still require delivery in the micromolar range [105,233]. Multimeric versions of existing CPPs have been reported to increase delivery, but methods for making multimeric CPPs are complex. In addition, conjugation of cationic CPPs can precipitate otherwise-stable proteins [234], and conjugating highly charged multimeric TAT/polyarginine could exacerbate this issue.

CPPs have been reported to inhibit antibody function, requiring cleavable linkers to liberate excess charge from IgGs and restore binding [117]. Furthermore, serum proteins are known to inhibit CPP uptake and alter subcellular localization [121,235]. Taken together, these factors bode poorly for in vivo pharmacokinetics and bioavailability of CPP-protein drugs, limiting their translational potential. This is further evidenced by the complete lack of FDA-approved CPP-based biologics. Recent studies combining endosomolytic assist peptides with CPPs have demonstrated significant improvements in CPP-mediated delivery regarding cytosolic access [127,131], but these technologies have not been extended beyond gene editing enzymes nor have they been tested for in vivo protein delivery.

Alternatively, bacterial toxins have shown high delivery rates with appropriate retargeting. Unfortunately, these methods are variable and highly cell type dependent. In addition, most bacterial systems require additional protein engineering steps to reduce toxicity. For example, the widely studied LF/PA system requires 2-component engineering of both the lethal factor and PA to abrogate cytotoxicity and promote efficient translocation. Another drawback of the Anthrax PA/LF_N_ platform is that protein cargo must be unfolded to pass through PA pores. Thus, cargo proteins with high thermodynamic stability are inefficiently translocated into the cytosol [147,236], and payload function could be lost if proteins cannot properly refold in the reducing cytosolic milieu. Finally, bacterial toxin components may trigger immune responses, further limiting their clinical application.

### Nanocarriers

Nanocarriers such as micelles, vesicles, solid nanoparticles, or supramolecular complexes offer many advantages compared to naked protein delivery including high loading capacity and protection from degradation. Still, nanoparticle-mediated delivery of proteins faces several challenges. Chief among these drawbacks is that nanoparticles, with relatively large sizes compared to most proteins, can exhibit poor tissue penetration. Notably, nanoparticle transport across the healthy, undisturbed epithelium is severely restricted, precluding their access to certain organs such as the brain. In some pathologies such as solid tumors and fibrosis, a dense extracellular matrix poses a second barrier to nanoparticle-mediated protein delivery [237]. Moreover, potential acute and chronic toxicities of nanoparticle components and their degradation products are constant concerns and must be mitigated during formulation development [238]. Nonetheless, methods to fine-tune the composition, size, and surface functionality of nanoparticles have provided a toolbox for addressing these biological hurdles through rational design, making nanomaterials an attractive method for protein drug delivery.

Notably, polymers benefit from an abundance of available functional groups that can be chemically grafted to grant nearly endless combinations of physiochemical properties. This flexibility has afforded researchers polymeric encapsulation of both native and modified proteins by a combination of electrostatic, coordination, and van der Waals interactions. However, synthesis of new, bespoke polymers is time-consuming, and new materials must be tested for biocompatibility. Finally, polymer:protein nanoparticles are generally more polydisperse than other nanocarriers including inorganic nanoparticles and LNPs. This is especially true for polymer–protein supramolecular complexes. Nanoparticle size heterogeneity could lead to unpredictable biodistribution and pharmacokinetics resulting in adverse effects.

Liposomes are easily produced but suffer from low protein encapsulation efficiency. Methods to improve protein packing into liposomes are harsh and can damage protein cargo. Alternatively, LNPs have demonstrated higher encapsulation efficiencies than liposomes. Moreover, cationic, ionizable and fusogenic lipid components can destabilize endosomal membranes and aid protein release into the cytosol. However, successful encapsulation of proteins within LNPs does require optimization. Firstly, for high loading efficiency, the protein cargo must be co-engineered along with the LNP constituents to promote encapsulation. In addition, cationic lipids commonly used in protein LNP formulations such as DOTAP, DOTMA, or DOSPA have documented cytotoxic effects in vivo via generation of reactive oxygen species [239]. To limit LNP-associated toxicities, clinically used protein LNPs should incorporate a higher proportion of ionizable lipids relative to cationic lipids. Methods to generate vast ionizable lipid libraries with distinct branching, saturation, and charge features are readily available [162,163,165]. However, synthesis and screening of these lipids for protein delivery remain time-consuming. Additional work is needed to establish the structure–function relationships governing efficient and stable encapsulation of protein cargo within LNPs.

Popular inorganic nanoparticles such as AuNPs [240] and SiNPs [241] are easily synthesized via well-established bulk methods. However, protein adsorption might cause cargo unfolding and functional loss. In addition, if the protein–nanoparticle interaction is weak, reliance on surface adsorption could risk premature cargo release in circulation. Furthermore, in many inorganic nanoparticle formulations, surface-bound proteins are exposed to the serum and are more susceptible to enzymatic degradation compared to encapsulated proteins. These materials may also exhibit poor biodegradability leading to protracted elimination. This has been shown to be the case for AuNPs [242].

VLPs have emerged as an exciting biological nanocarrier for protein delivery. They are able to mimic the natural ability of viral vectors to infect host cells without inducing permanent genetic changes. However, several factors for successful VLP production and deployment should be considered. Firstly, the functionality of some cargo may be lost upon fusion to viral proteins due to misfolding of cargo-Gag/Gag-Pol protein chimeras. Hence, for proteins intolerant to Gag/Gag-Pol fusion, efficient proteolytic cleavage is necessary for activation. In addition, viral titers invariably suffer from fusion of exogenous genes to Gag/Gag-pol. Viral capsid, matrix, and envelope proteins present in VLPs can also trigger unintended immune response in vivo, limiting the clinical scope of this modality. Importantly, because cargo-virus components must be expressed in mammalian cells, cytotoxic proteins cannot be packaged by the host cell for VLP-mediated delivery. For example, Kaczmarczyk et al. [216] was unable to package whole caspase into VLPs and had to implement a split system by simultaneous delivery of caspase fragments in separate VLPs.

In recent years, cell-derived exosomes have emerged as biocompatible and stable vehicles for cytosolic delivery of various cargo including proteins. Since exosomes are produced naturally by all tissues and orchestrate cell-to-cell communication across all organ systems, they have a very low risk of immunogenicity. Unfortunately, many of their drawbacks also stem from their biological origins. Notably, exosomes suffer from low production yields and complicated harvesting protocols that require lengthy ultracentrifugation steps. Following exosome harvesting, proteins must be loaded into the vesicles, further increasing manufacturing time. Systems like EXPLORs combine protein loading with exosome biogenesis for an all-in-one production method. However, as therapeutic proteins may also be toxic to the mammalian producer cells, this method is precluded from some cargo including apoptosis-inducing proteins. In addition, cargo loading efficiency by EXPLORs is low at 1 to 2 proteins per exosome [224]. This hurdle can be circumvented by electroporation, a standard technique affording ~50% loading efficiency [222]. Thus, for most therapeutic applications, protein loading of harvested exosomes remains the optimal method for exosome-mediated delivery. Another potential drawback for cell-derived exosomes is that they are inherently less “pure” than synthetic nanocarriers. In addition to the intended payload, exosomes contain components of the production cell including RNA, DNA, and endogenous proteins. Finally, unmodified exosomes tend to be trapped within endosomes, and although cytosolic access can be enhanced by coating the surface with cationic lipids or fusogenic peptides [243], this step adds another layer of complexity to exosome-mediated delivery.

### Maintaining protein folding and function

A major concern for any protein delivery system is preserving cargo function through the various stages of purification, conjugation/modification, and delivery processes. In contrast to DNA or RNA, the proper tertiary, or sometimes quaternary, structure of proteins must be preserved during encapsulation and delivery. Methods requiring prolonged exposure of proteins to extreme pH conditions, salt concentrations, or organic solvents may denature protein payloads, especially fragile enzymes. Fusion of highly charged or hydrophobic CPPs may also dramatically alter the folding, expression, and activity of desired therapeutic proteins. In addition, repeated freeze–thaw cycles, previously implemented for liposome encapsulation [156,157], could irreversibly damage payloads. Thus, fundamental research to better understand how proteins interact with peptides, polymers, lipids, detergents, and solvents is necessary to improve cargo stability and functionality.

LNPs provide a promising alternative to liposomes when encapsulating negatively charged proteins, but still present some formulation challenges. Specifically, LNPs are typically formed under acidic pH conditions (pH 3 to 5), as the ionizable lipids are protonated at pH values below their pKa and facilitate complexation with anionic nucleic acids. However, if the cargo protein is pH-sensitive, acidic buffers can denature cargo during the encapsulation process, abolishing activity and/or inducing aggregation. Neutral buffers can be used to avoid this issue, but a higher percentage of cationic lipids (such as DOTAP) must be incorporated into the LNP formulation to promote protein encapsulation [173]. However, increasing the amount of DOTAP can result in a trade-off in cytotoxicity due to reactive oxygen species produced by cationic lipids. Thus, the optimal LNP formulation must consider the pH tolerance of the protein in order to balance both encapsulation with cytotoxicity. Choosing an appropriate ionizable lipid with an apparent pKa greater than the isoelectric point of the cargo can alleviate this issue. In addition, anionic charge modification decreases proteins’ isoelectric point, making them more tolerant to acidic conditions. We have shown that some ionizable lipids can encapsulate proteins at near-neutral pH conditions (pH 6.0 to 6.5) for high-efficiency cytosolic delivery with reduced toxicity [179]. Together, these results highlight the need to screen ionizable LNP formulations for each protein payload.

Attaching too many chemical groups to cargo proteins including antibodies can lead to immediate aggregation. Even if solubility is maintained, plasma stability can be impacted, and binding affinity can be hampered by steric blocking [244]. One way to avoid clashes with active domains is to site-specifically label proteins. Our group has developed 2 enzymatic methods: sortase-tag expressed protein ligation [245] and proximity-based sortase-mediated ligation [246] for one-step purification–bioconjugation of proteins and site-specific labeling at the C-terminus. For IgG conjugation at the Fc region, our lab frequently uses light-activatable antibody binding-domains derived from protein Z [247] or protein G [176]. Other chemical and chemoenzymatic methods for site-specific antibody labeling are also available [84,244]. In addition, using a large carrier such as a dendrimer can enable conjugation of multiple payloads molecules per antibody without the affinity loss associated with conjugating excess payload directly to antibodies [248].

Charge modification by indiscriminate conjugation to surface residues may negatively impact protein function and should be avoided. Controlled, site-specific methods for negative charge introduction are therefore favored to mitigate protein inactivation while permitting electrostatic interaction with charged lipids. This strategy of fusing a negative “carrier” peptide/protein rather than directly modifying the cargo has successfully been widely utilized for efficient cytosolic protein delivery while preserving functionality [166,167,170,177–179,181,202]. Indeed, we have found that some highly stable protein scaffolds, such as DARPins, are amenable to charge-conjugation and exhibit no decrease in binding affinity following charge fusion. However, others have reported decreased binding affinity of other scaffolds including the popular nanobody format following fusion of cationic CPPs [135,249]. The extent of affinity reduction is likely dependent on the binder scaffold itself and fusion protein design. In some applications such as bioPROTAC delivery, degradation functionality may be completely unaffected even if binding is weakened if the affinity remains above a threshold for target engagement and ubiquitination [42,72].

For protein cargo containing disulfide bonds, namely, full-length antibodies, the reducing cytosolic environment could potentially destabilize their structure upon intracellular delivery. While we and many others have successfully delivered antibodies into cells while retaining cytosolic activity, it is possible that some IgGs or other disulfide-stabilized proteins are more sensitive to glutathione-mediated reduction. New protein engineering strategies to either improve cargo resistance or eliminate the need for cysteine residues altogether would greatly benefit the field. Foundational research on cytosolically folded intrabodies has provided valuable insights into antibody stability in the cytosolic milieu [250–252], and additional work is necessary to fully reveal mechanisms of protein unfolding and degradation as well as offer strategies toward improving protein cargo themselves.

### Readouts of cytosolic delivery

Accurate assessment of cytosolic protein delivery is critical for the development and refinement of new methods and technologies. Sensitive quantitation of compartment-specific delivery is valuable, as many therapeutic proteins exhibit activity only in the cytosol. Confounding readouts can be caused by proteins trapped on the outer membrane or within endosomes, which may lead to overestimation of performance. Despite the need for a reliable and quantitative delivery assay, few assays offering high sensitivity and cytosolic discrimination exist.

Immunoblotting is a well-established and direct method for detecting cytosolic proteins, but it suffers from many limitations as a protein delivery assay. Critically, Western blotting is semi-quantitative, requiring careful optimization to stay within the linear range. In addition, immunoblotting is low-throughput and time-consuming. Direct detection of unmodified proteins by Western blotting is also limited by available primary antibodies against the protein, and even when antibodies are available, standard protocols cannot differentiate between cytosolic and endosomal fractions. Some of these issues have been addressed to improve immunoblotting for protein delivery. For example, epitope tags including HA and FLAG can be appended to proteins for facile detection using commercial anti-HA and anti-FLAG primary antibodies. Verdurmen et al. [147] fused an Avi-tag to proteins and delivered them into biotin ligase-expressing cells. As biotin ligase is localized to the cytosol, the Avi tag is only biotinylated upon endosomal escape of the cargo protein and can be detected with either anti-biotin antibodies or streptavidin conjugates. Despite these improvements, Western blots remain low-throughput and exhibit low reproducibility. For these reasons, immunoblotting is generally a poor assay for detecting and quantifying exogenously delivered protein.

Cre recombinase is an enzyme that catalyzes recombination of DNA sequences flanked by 34-base-pair LoxP recognition sequences, known as “floxed” genes. Cre recombinase can be used as a model protein to validate cytosolic delivery, since its activity depends on cytosolic access and subsequent nuclear translocation. Multiple studies have shown protein delivery using genetically encoded fluorescent reporters downstream of floxed stop cassettes [166,167,170]. In addition to strictly reporting cytosolic delivery, Cre-Lox systems are sensitive, as Cre-mediated excision is permanent. Just a few active molecules of Cre can drive potent expression of a fluorescent marker when placed downstream of a strong promoter. While Cre-lox-based readouts offer high sensitivity, the assay can also be misleading. Since Cre-lox systems fundamentally work by “turning-on” gene expression, readout is greatly amplified and may overestimate protein delivery efficiency.

Fluorescence is another popular method for detecting intracellular proteins. For fluorescence-based detection, cargo proteins can be easily labeled with dyes using commercially available kits. Alternatively, naturally fluorescent proteins, including GFP and its variants, can be used as model cargo for a given delivery system. However, GFP delivery may not represent cytosolic delivery of other therapeutic proteins with varying structure and surface charge.

Due to the simplicity of fluorescence readouts by either flow cytometry or microscopy, numerous studies have validated protein delivery using GFP cargo or dye-labeled proteins. While easily implemented, most fluorescent proteins are detectable regardless of cellular localization, so proteins trapped within endosomes or adsorbed onto cell membranes cannot be easily differentiated from cytosolically delivered proteins. Some strategies have been introduced to overcome these limitations. In some cases, surface binding of fluorescent proteins can be ruled out using an appropriate quenching dye. For example, trypan blue can be used to quench surface-bound GFPs and FITC [197]. With microscopy, proteins trapped within endosome–lysosomes can usually be qualitatively distinguished as fluorescent puncta, but with more quantitative methods such as flow cytometry, this distinction cannot be made. Finally, pH-sensitive dyes have been employed to track endosomal escape via conditional fluorescence in acidic endosomes [253], but this method produces variable results, since endosomal pH varies throughout the endosome maturation process. Importantly, regardless of any experimental modification discussed above, constitutively fluorescent proteins and dyes cannot be used for live-animal in vivo delivery assays, as there is no mechanism to differentiate intracellular from extracellular proteins without harvesting tissues for further processing and inspection.

To overcome the limitations of standard fluorophores, split fluorescent proteins can be used instead. In one popular split fluorescent protein system, GFP is divided into 2 fragments: the larger GFP(1–10) and the 11th beta strand, S11 [254]. These 2 fragments exhibit weak fluorescence on their own, but upon complementation, the 2 fragments reconstitute a fluorescent GFP molecule. In a typical split GFP delivery assay, the cargo protein is fused with the S11 peptide, as its small size (16 amino acids) is unlikely to perturb folding or function. The S11-tagged protein is then delivered into a reporter cell line expressing GFP(1–10), and complementation can be detected by microscopy or flow cytometry. Unlike conventional fluorophores, split GFP detection is conditional upon both intracellular delivery and endosomal escape, since GFP(1–10) is localized to the cytosol. These features make split fluorescent protein assays highly attractive for validating cytosolic protein delivery and to measure delivery efficiency. When designing split GFP assays, some drawbacks must be considered. Firstly, the limit of detection for split GFP has been reported to be in the nanomolar range [255]. This can limit the utility of split GFP for some proteins including high-affinity antibodies and enzymes with therapeutic activity at picomolar concentrations. Furthermore, GFP must undergo a relatively slow maturation process before it can be detected, leading to low temporal resolution in the first few hours post-delivery.

**Fig. 7.**
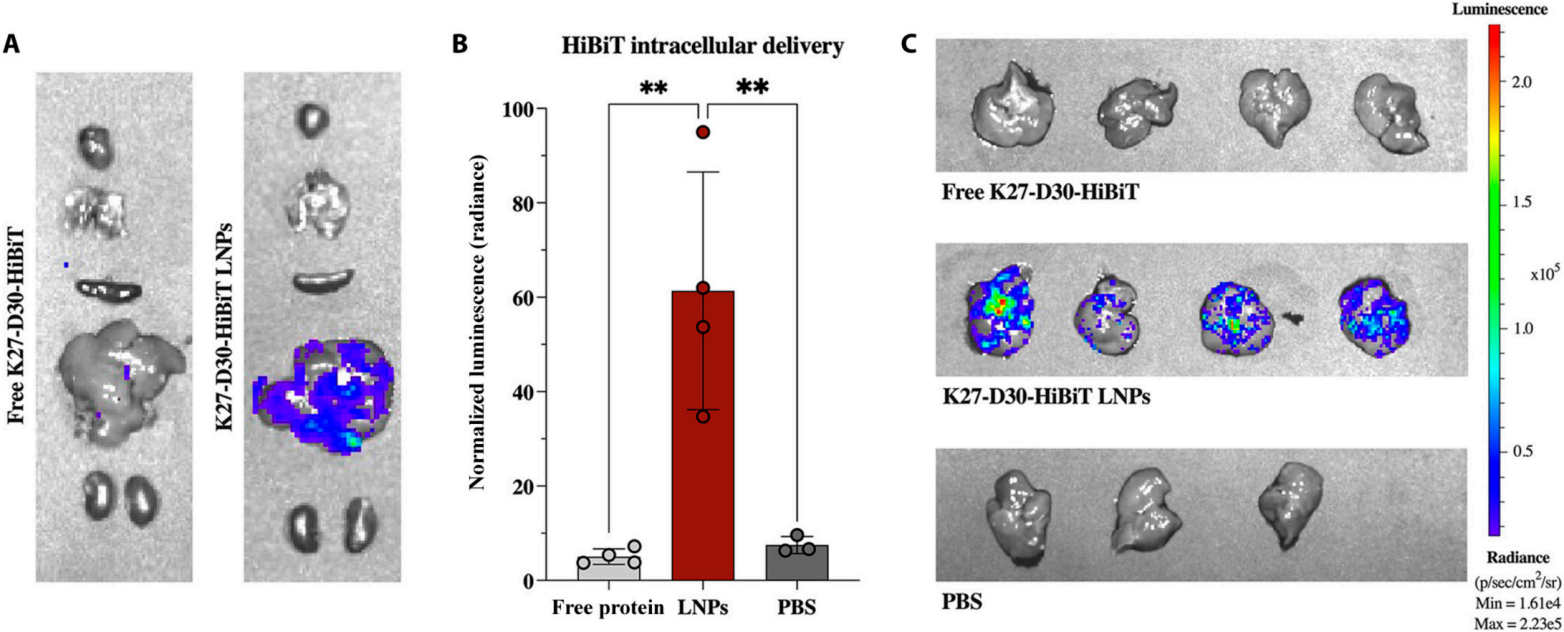
Intracellular delivery of LNP:protein in vivo. (A) Mice transfected with an LgBiT transposon reporter were injected I.V. with either LNP:DARPinK27-D30-HiBiT or free DARPinK27-D30-HiBiT. Bioluminescent imaging of organs ex vivo detected NanoLuc activity in the LNP treatment group only. (B) Quantitation of NanoLuc activity in livers from mice left untreated, injected with free protein, or injected with LNP:protein. (C) Ex vivo bioluminescent images of livers quantified in B. Reprinted (adapted) with permission from Haley et al. [179]. Copyright 2023 American Chemical Society.

Split luminescent reporters such as the split nanoluciferase (NanoLuc) system can overcome lower sensitivity split fluorescent proteins. In this method, reporter cells are engineered to stably express LgBiT, an 18-kDa fragment of NanoLuc, and protein cargo are tagged with the 11-amino-acid HiBiT peptide. Once delivered into the cytosol, HiBiT binds to LgBiT with high affinity (0.7 nM) to reconstitute NanoLuc, a bright luminescent reporter [256]. Cytosolic delivery assays based on split NanoLuc have demonstrated higher sensitivity compared to split GFP with detection possible at concentrations as low as 5 pM [255]. Additionally, unlike split GFP, split NanoLuc does not require fluorophore maturation, and cytosolic delivery can be detected almost immediately following LgBiT/HiBiT complementation. These features make the LgBiT/HiBiT system an attractive method for rapid and sensitive quantitation of cytosolically delivered proteins, and we recently adopted this technology for the detection of cytosolically delivered proteins in mice (Fig. 7).

Unfortunately, for live-animal in vivo imaging, both split fluorescent proteins and split NanoLuc are disadvantaged. Critically, the excitation and emission wavelengths for GFP and NanoLuc are strongly absorbed by tissues. Split fluorescent proteins in the NIR range have been developed for tissue penetration, but many of these reporters are designed to study PPIs and do not readily self-associate. To extend NanoLuc for deep-tissue imaging, Chu et al. [257] developed Antares, a fusion protein comprising NanoLuc sandwiched between 2 optimized orange fluorescent proteins (CyOFP1). In a process known as bioluminescence resonance energy transfer (BRET), CyOFP1 absorbs blue light emitted by NanoLuc and its substrate, converting it into orange–red light with a peak wavelength of 589 nm. This red-shifted signal better penetrates tissues and enables live-animal imaging with a bright luminescent reporter. Interestingly, this raises the possibility of adapting Antares for live animal detection of cytosolic protein delivery by replacing NanoLuc with LgBiT. Protein cargo tagged with HiBiT can bind to CyOFP1-LgBiT fusions expressed in reporter cells for BRET-mediated detection. Recently, a version of such a split-Antares protein was reported for in vivo small-molecule sensing [258]. Alternatively, if a delivery system is agnostic to the identity of the cargo, Cre recombinase may be suitable for tracking in vivo cytosolic delivery by activating firefly luciferase expression for standard bioluminescent imaging.

### Tissue targeting

To decrease toxicity and improve therapeutic benefit, cytosolic delivery systems must be able to direct proteins to the appropriate organs and/or cell type. For CPP, bacterial toxin, and other “naked” delivery systems, proteins can be genetically fused with targeting domains. Many tumor-specific peptides have been reported and can be appended to existing CPP-bearing proteins. For example, the cell-penetrating anti-RAS antibodies RT11 and inRas37 were fused with integrin-binding peptides for cancer targeting [125,126]. An EpCAM-binding variant of inRas37 for treatment of pancreatic cancers was developed by simply swapping the integrin-binding peptide with an EpCAM-recognizing cyclic peptide [259]. Optimization of this EpCAM-targeting peptide resulted in low nanomolar affinity and enhanced accumulation in EpCAM-expressing tumors compared to the original integrin αvβ3/αvβ5 targeting peptides [260]. In addition, hybrid “targeting penetrating peptides” with dual tumor targeting and internalization functions have been published [261]. Tumor targeting is also possible through conditionally active CPPs. These activatable CPPs are tethered to an inhibitory domain via peptide linkers sensitive to MMP, cathepsin, or urokinase cleavage [262]. Since these proteases are upregulated in many cancers but not in healthy tissues, enzymatic processing of the linker and subsequent release of the inhibitory domain is site-specific, resulting in preferential tumor uptake.

Targeting with Anthrax-based platforms has been achieved by mutating the furin cleavage site of PA to tumor-specific protease sequences [144]. PA-mediated LF/EF uptake is normally activated by proteolytic processing at furin-recognition motif. Thus, replacing this furin-cleavage site with MMP or urokinase sensitive sequences can confer tumor specificity in a similar manner to protease-activatable CPPs. More commonly, targeting domains such as growth factors [145], affibodies [146], and DARPins [147,148,263] are genetically fused to PA to actively redirect PA and, consequently, LF-cargo to specific cells of interest.

Targeted nanoparticles have been widely studied for tissue-specific drug delivery. A standard method to produce targeted nanoparticles is by functionalizing nanomaterial surfaces with target-specific binders including small molecules, peptides, DNA aptamers, and folded proteins. Known as “active targeting”, this approach seeks to direct nanoparticles to specific cells and tissues via overexpressed or unique surface receptors. Proteins such as IgGs or small protein scaffolds are commonly employed for actively targeted nanoparticles, because they are readily available and demonstrate high affinity and specificity. In one study, PECAM-1 specific antibodies were conjugated to mRNA-loaded LNPs and increased gene expression in lungs by 50-fold compared to isotype control [264]. Recently, conjugation of either anti-CD4 [265] or anti-CD5 [266] antibodies to mRNA LNPs has enabled T cell engineering in vivo. Antibody and peptide conjugation of gold [267] and silica nanoparticles [268] have been thoroughly investigated for tumor targeting. In addition, inorganic nanoparticles are frequently modified with folic acid to target folate receptors commonly overexpressed in epithelial cancers [269].

It is also possible to modulate biodistribution without attaching binding ligands to nanoparticles through passive approaches based on tuning nanoparticle physical properties. Broadly speaking, the basic principles governing nanoparticle biodistribution are well established. Notably, liver accumulation is known to correlate positively with particle size. This trend holds true for many classes of nanoparticles including AuNPs [270], SiNPs [271], and polymeric nanoparticles [272] Conversely, smaller nanoparticles (<5 nm) are typically excreted in the urine. In nearly all cases, coating nanoparticles with polyethylene glycol (PEG) confers “stealth” properties for prolonged circulation and evasion of excretion.

With the successful launch of multiple LNP-delivered RNA drugs in the past few years, many have sought to modify LNP tropism to expand their therapeutic utility. For example, preferential organ accumulation of systemically administered ionizable LNPs can be modulated by the amount of cationic lipids used during formulation [172,173]. Moreover, differential delivery of RNA LNPs to cell types within the liver is also possible by adjusting the proportion of PEG-lipid [273]. Multiple reports have also demonstrated that both charge and size of LNPs affect their tropism and delivery to different immune cell subtypes [274].

For bioinspired nanocarriers such as cell-derived exosomes, particle properties are less easily manipulated, as their composition is already defined at the time of harvesting. The protein and lipid composition of exosomes’ outer membrane largely determines its specificity. Exosomes are preferentially taken up by target cells with similar membrane composition to the production cell. Thus, one strategy for targeting exosomes to the correct tissue is to isolate them from the same types of cells as the target.

## Conclusion

Despite the vast potential of cytosolically delivered proteins, to date, no FDA-approved drugs utilizing this modality exists. Intracellular protein delivery could unlock safer and more potent drugs for a broad range of diseases. A major hurdle for cytosolic protein drugs is a lack of scalable and efficient methods for intracellular delivery. Initially, CPPs garnered much attention as potential cell transduction domains, but many CPP-based delivery strategies are burdened by poor internalization efficiencies and/or endosomal escape. However, recent findings renew the promise of CPPs, especially for Cas-mediated gene editing.

Researchers have also turned to nanocarriers as a means for protein transfection. Nanoparticles offer many attractive properties such as high loading capacity, functional tunability, decreased immune responses, and protection from enzymatic degradation. A universal nanoparticle approach for cytosolic protein delivery, like those that exist for nucleic acids, would revolutionize biotechnology and human health.

It remains unclear whether unmodified protein cargo can be delivered at the same levels as modified cargo. Therapeutic proteins differ radically in size, structure, surface charge, hydrophilicity/hydrophobicity, and oligomeric state. All of these properties strongly influence protein interactions with delivery agents. In addition, protein delivery is known to depend on the interaction strength between cargo and carrier [275]; thus, enforcing an anionic charge either by chemical conjugation or by genetic fusion is an attractive and reliable strategy for improving protein cargo loading.

In summary, many strategies for cytosolic protein delivery have been proposed over the past few decades. In addition, continued research into biological transport systems remains a key pillar for the development of new technologies. Among the ever-growing approaches to cytosolic delivery, each method offers unique benefits as well as disadvantages depending on the function, structure, and physical properties of the protein drug. Moreover, the therapeutic context for a particular protein is highly important and can dictate the optimal modality. Clinical applications of intracellular protein delivery might necessitate a diverse toolbox of complimentary and disease-specific techniques. Key challenges facing the protein delivery field are low encapsulation efficiencies and endosomal entrapment. Moreover, tissue-specific targeting of intracellularly delivered proteins has not been thoroughly explored for protein delivery systems. In parallel, assays for protein delivery are urgently needed, so researchers can benchmark new delivery approaches. Addressing these concerns is critical to fully realizing the therapeutic potential of protein-based drugs.
